# Two New Sesquiterpenoids and a New Shikimic Acid Metabolite from Mangrove Sediment-Derived Fungus *Roussoella* sp. SCSIO 41427

**DOI:** 10.3390/md22030103

**Published:** 2024-02-23

**Authors:** Zimin Xiao, Jian Cai, Ting Chen, Yilin Wang, Yixin Chen, Yongyan Zhu, Chunmei Chen, Bin Yang, Xuefeng Zhou, Huaming Tao

**Affiliations:** 1 Guangdong Provincial Key Laboratory of Chinese Medicine Pharmaceutics, School of Traditional Chinese Medicine, Southern Medical University, Guangzhou 510515, China; 15917491112@163.com (Z.X.); 13676126834@163.com (Y.C.); yongyanzhu0521@163.com (Y.Z.); 2 CAS Key Laboratory of Tropical Marine Bio-Resources and Ecology, Guangdong Key Laboratory of Marine Materia Medica, South China Sea Institute of Oceanology, Chinese Academy of Sciences, Guangzhou 510301, China; caijian19@mails.ucas.ac.cn (J.C.); chenchunmei18@mails.ucas.ac.cn (C.C.); yangbin@scsio.ac.cn (B.Y.); xfzhou@scsio.ac.cn (X.Z.); 3 School of Stomatology, Southern Medical University, Guangzhou 510515, China; chent@smu.edu.cn (T.C.); FOURCWATER@outlook.com (Y.W.)

**Keywords:** mangrove sediment-derived fungi, sesquiterpenoid, IL-1β, anti-inflammatory

## Abstract

Two new sesquiterpenoid derivatives, elgonenes M (**1**) and N (**2**), and a new shikimic acid metabolite, methyl 5-*O*-acetyl-5-*epi*-shikimate (**3**), were isolated from the mangrove sediment-derived fungus *Roussoella* sp. SCSIO 41427 together with fourteen known compounds (**4**–**17**). The planar structures were elucidated through nuclear magnetic resonance (NMR) and mass spectroscopic (MS) analyses. The relative configurations of **1**–**3** were ascertained by NOESY experiments, while their absolute configurations were determined by electronic circular dichroism (ECD) calculation. Elgonene M (**1**) exhibited inhibition of interleukin-1β (IL-1β) mRNA, a pro-inflammatory cytokine, at a concentration of 5 μM, with an inhibitory ratio of 31.14%. On the other hand, elgonene N (**2**) demonstrated inhibition at a concentration of 20 μM, with inhibitory ratios of 27.57%.

## 1. Introduction

As a defensive response to injury, inflammation is not always harmful; however, excessive expression of inflammatory mediators can lead to immune system dysregulation, resulting in inflammatory diseases [1]. Currently, the most widely used anti-inflammatory drugs worldwide are nonsteroidal anti-inflammatory drugs (NSAIDs), but they come with serious gastrointestinal side effects and cardiovascular risks [2]. Therefore, exploring new anti-inflammatory drugs is crucial research. Scientists are actively investigating natural sources, including marine organisms, plants, and microorganisms, in the quest for new therapeutic agents with potent anti-inflammatory properties and improved safety profiles.

The ocean is a treasure trove of resources, and the search for new natural products from the marine environment for drug development has become an international research hotspot. Statistics show that, in the year 2021 alone, researchers discovered 1425 new compounds with a wide range of biological activities from marine-derived organisms [3]. Among these, mangrove sediment-derived microorganisms (MSMs) are an important source of various natural products. As of 2021, researchers have isolated and identified 519 new natural products from MSMs, with 57% of these compounds originating from fungi and exhibiting broad and effective biological activities. These compounds from marine-derived fungi have shown promising antimicrobial properties against various pathogens, significant anticancer potential, anti-inflammatory effects, antioxidant activity, and additional biological activities, such as antiviral, antiparasitic, and immunomodulatory effects [4]. The discovery of these 519 new natural products from MSMs, particularly with a significant proportion originating from fungi, highlights the immense potential of marine ecosystems as a source of bioactive compounds. The exploration of marine-derived fungi and other MSMs continues to be a fruitful area of research, offering new avenues for drug discovery and development. By harnessing the diverse chemical structures and biological activities of these natural products, scientists aim to address various health challenges and improve the well-being of humans.

*Roussoella* sp. SCSIO 41427 belongs to the Ascomycota phylum, and, in previous research reports, numerous structurally novel and biologically active secondary metabolites have been discovered from strains of this kind [5] (for instance, from *Roussoella hysterioides* KT1651, tetracyclic diterpene fusicoccanes, roussoellols A and B, with unique bent structural frameworks [6]. Additionally, a novel dehydroacetic acid derivative, roussoellenic acid, isolated from *Roussoella* sp. (MFLUCC 17-2059), displayed excellent inhibitory activity against biofilm formation in *Staphylococcus aureus* [7].

**Figure 1:**
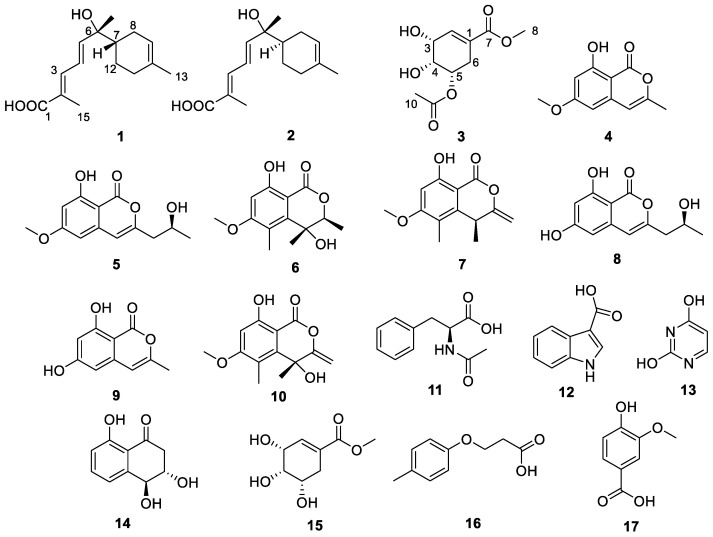
Structures of compounds **1**–**17**.

In our previous research, we successfully isolated a multitude of structurally novel compounds from marine-derived fungi. These compounds encompass a variety of new polyketides, alkaloids, and other metabolites, each showcasing a diverse array of noteworthy biological activities. Notably, these compounds demonstrate promising anti-inflammatory, antifungal, and antitumor properties [8,9,10]. In this study, we extracted, isolated, and identified seventeen compounds (Figure 1) from the rice fermentation products of the strain *Roussoella* sp. SCSIO 41427, sourced from mangrove sediment. Among them are two new sesquiterpenoids, elgonenes M (**1**) and N (**2**), one new natural product (**3**) (previously reported synthetically [11]), along with seven known isocoumarins derivatives (**4**–**10**) and some other known compounds (**11**–**17**). Notably, elgonenes M (**1**) and N (**2**) demonstrated a reduction in the expression of endogenous inflammatory factor IL-1β mRNA at concentrations of 5 μM and 20 μM, with inhibition rates of 31.14% and 27.57%, respectively. This paper focuses on the isolation, structural elucidation, and details of the biological activities of these compounds.

## 2. Results and Discussion

### 2.1. Structural Determination

**Figure 2:**
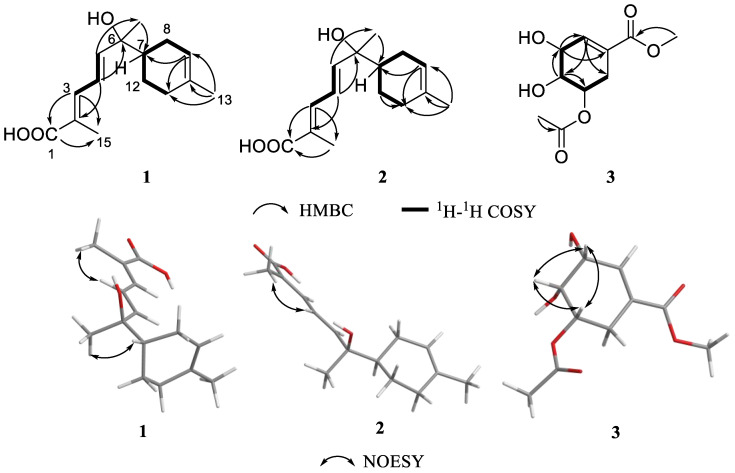
Key ^1^H-^1^H COSY, HMBC, and NOESY correlations of **1**–**3**.

Compound **1** was obtained as a pale yellow oil, and its molecular formula of C_15_H_22_O_3_ was deduced from the negative HRESIMS ion peak at *m*/*z* 249.1499 [M-H]^−^ (calculated for C_15_H_21_O_3_^−^, 249.1496), implying five degrees of hydrogen deficiency. The ^1^H NMR data (Table 1) showed three methyls at *δ*_H_ 1.33 (s, H_3_-14), 1.64 (s, H_3_-13), and 1.96 (s, H_3_-15); three methylenes at *δ*_H_ 1.81/2.03 (H_2_-8), 1.99 (m, H_2_-11), and 1.24/1.90 (H_2_-12); and five olefinic protons at 7.29 (d, *J* = 11.4 Hz, H-3), 6.59 (dd, *J* = 11.4, 15.1 Hz, H-4), 6.17 (d, *J* = 15.1 Hz, H-5), 1.63 (m, H-7), and 5.36 (m, H-9). Analysis of the ^13^C, DEPT135 and HSQC NMR spectra displayed 15 carbon signals, including one carbonyl carbons at *δ*_C_ 173.5 (C-1); two olefinic tertiary carbons at *δ*_C_ 126.1 (C-2), and 134.2 (C-10); four olefinic methine carbons at *δ*_C_ 140.1 (C-3), 123.5 (C-4), 148.0 (C-5), and 120.3 (C-9); three methyl carbons at *δ*_C_ 23.5 (C-13), 26.1 (C-14), and 12.6 (C-15); three methylene carbons at 26.9 (C-8,), 30.8 (C-11), and 23.6 (C-12); and two methine carbon (including one oxygenated) at 75.3 (C-6) and 44.3 (C-7). The ^1^H-^1^H COSY correlations (Figure 2) of H_2_-11/H_2_-12/H_2_-7/H_2_-8/H-9 and the HMBC correlations from H_3_-13 to C-9, C-10, and C-11 revealed a six-membered ring with a methyl at C-10. A chain system with hydroxyl and carboxyl groups was confirmed by the ^1^H-^1^H COSY correlations of H-3/H-4/H-5 and the HMBC correlations from H-2 to C-1 and C-4, as well as from H-4 to H-6 and from H-5 to H_3_-14. The HMBC correlation from H_3_-14 to C-7 suggested that methine carbon C-6 at the end of the chain was located at C-7 on the ring. Furthermore, the NMR data indicated that the planar structure of **1** was similar to that of the known compound, sesquiterpene elgonene D [12]. Also, Compound **2** was obtained as a pale yellow oil, and its molecular formula of C_15_H_22_O_3_ was deduced from the negative HRESIMS ion peak at *m*/*z* 249.1498 [M-H]^−^ (calculated for C_15_H_21_O_3_^−^, 249.1496). The NMR spectroscopic data (Table 1) comparison between **2** and **1** revealed that they possess identical planar structures.

**Figure 3:**
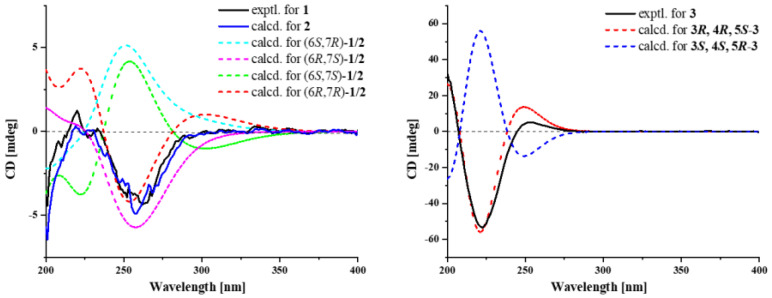
Experimental and calculational ECD spectrum of **1**–**3**.

As for their configuration, Δ^2,3^ and Δ^4,5^ double bonds in **1** and **2** were both deduced as *E* by the NOESY correlation of H_3_-15/H-3 (Figure 2) and the large coupling constant *J*_H-4/H-5_ = 15.1/15.2 Hz. Due to the consistent trends observed in their experimental CD curves, the ECD calculations of **1**/**2** (Figure 3) indicated that the configuration was established as 6*R*,7*S*/6*R*,7*R*. Thus, compounds **1** and **2** are a pair of diastereomers, with both having an *R* configuration of C-6. To differentiate between the diastereomers **1** and **2**, a detailed conformational analysis was performed. The dominant conformation of the 6*R*,7*S* stereoisomer was characterized by a close spatial proximity between H_3_-14 and H-7, indicating the presence of a NOESY effect of H_3_-14/H-7 (Figure 2). Compared with the NMR experimental data, the NOESY correlation of H_3_-14/H-7 in **1** suggested its absolute configuration as 6*R*,7*S*. Similarly, the absence of a NOESY correlation of H_3_-14/H-7 in **2** suggested its absolute configuration as 6*R*,7*R*. Therefore, the gross structures, as depicted in Figure 1, were constructed and have been designated as elgonenes M (**1**) and N (**2**).

Compound **3** was isolated as pale yellow oil, and its molecular formula was determined as C_10_H_14_O_6_ by HRESIMS ion peak at *m*/*z* 231.0870 [M+H]^+^ (calculated for C_10_H_15_O_6_^+^, 231.0863), corresponding to four indices of hydrogen deficiency. The ^1^H NMR spectrum exhibited two methyl singlets at *δ*_H_ 2.10 (H_3_-10) and 3.76 (H_3_-8). Analysis of the ^13^C NMR (Table 1) and DEPT135 NMR spectra suggested the presence of three quaternary carbons, one CH_2_ group, including two carbonyl/ester-bearing quaternary carbons at *δ*_C_ 168.1 (C-7) and 172.3 (C-9), and one double-bond-bearing quaternary carbon at *δ*_C_ 129.2 (C-1). Additionally, the ^1^H NMR and HSQC data indicated the presence of four CH groups, including three oxygen-bearing CHs at *δ*_C_ 69.2 (C-3), 69.7 (C-4), and 72.4 (C-5), and one double-bond-bearing CH at *δ*_C_ 140.5 (C-2). The cyclohexene ring was determined through HMBC correlations from H-2 to C-4/C-6, H-3 to C-1/C-2, H-4 to C-2/C-3/C-5/C-6, and H_2_-6 to C-1/C-2/C-4/C-5. The structural elucidation involved establishing two side chains through HMBC correlations (Figure 2) from H_3_-8 to C-1/C-7 and H_3_-10 to C-5/C-9. The connection between the side chain structure and the cyclohexene ring was revealed through HMBC correlations from H-2/H_2_-6 to C-7, H-5 to C-9/C-10. Furthermore, ^1^H-^1^H COSY correlations between H-2/H-3/H-4/H-5/H-6 confirmed the cyclohexyl structure. The above NMR data indicated that the structural skeleton of **3** was identical to that of a synthesized compound, methyl 5-*O*-acetyl-5-*epi*-shikimate [11]. The NOESY correlations of H-3/H-5, H-3/H-4, and H-4/H-5 supposed that the relative configuration of **3** was rel-(3*R*, 4*R*, 5*S*). Finally, its absolute configuration was determined to be 3*R*, 4*R*, 5*S* through the ECD calculations and specific rotation compared with **15** (Figure 3).

By comparing their physicochemical properties and spectroscopic data with the reported literature values, other known compounds were determined. Compounds present in SCSIO 41427 were 8-hydroxy-6-methoxy-3-methyl-1*H*-isochromen-1-one (**4**) [13], (*S*)-8-hydroxy-3-(2-hydroxypropyl)-6-methoxy-1*H*-isochromen-1-one (**5**) [14], (3*S*,4*R*)-4,8-dihydroxy-6-methoxy-3,4,5-trimethylisochroman-1-one (**6**) [15], (*S*)-8-hydroxy-6-methoxy-4,5-dimethyl-3-methyleneisochroman-1-one (**7**) [16], (S)-6,8-dihydroxy-3-(2-hydroxypropyl)-1H-isochromen-1-one (**8**) [17], 6,8-dihydroxy-3-methyl-1*H*-isochromen-1-one (**9**) [18], 4,8-dihydroxy-6-methoxy-4,5-dimethyl-3-methyleneisochroman-1-one (**10**) [16], acetyl-*L*-phenylalanine (**11**) [19], 1*H*-indole-3-carboxylic acid (**12**) [20], pyrimidine-2,4-diol (**13**) [21], trans-3,4-dihydro-3,4,8-trihydroxynaphthalen-1(2*H*)-one (**14**) [22], methyl 5-*epi*-Shikimate (**15**) [11], 3-(*p*-tolyloxy)propanoic acid (**16**) [23], 4-hydroxy-3-methoxybenzoic acid (**17**) [24], related physicochemical and spectroscopic data shown in Supplementary Materials.

### 2.2. Bioactive Assay

**Figure 4:**
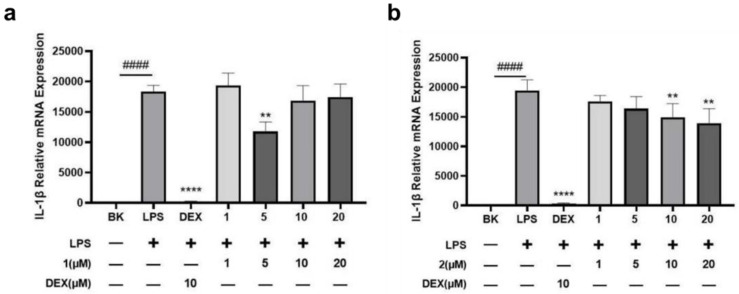
Inhibitory activity of **1** (**a**) and **2** (**b**) on IL-1β mRNA expression in LPS-induced macrophages. where values are expressed as mean ± standard deviation, *n* ≥ 3; #### *p* < 0.0001 for LPS-stimulated model group vs. blank control group; ** *p* < 0.01, **** *p* < 0.0001 for treatment groups vs. LPS-stimulated model group.

Most acute and chronic non-infectious inflammatory diseases are associated with the pro-inflammatory cytokine interleukin-1β (IL-1β), and clinical studies have proven that blocking IL-1β can effectively resolve inflammation [25]. In order to investigate the anti-inflammatory effects of the compounds, an inflammation model was established using lipopolysaccharide (LPS)-stimulated C57BL/6 mouse primary bone marrow-derived macrophages. Dexamethasone, a well-known anti-inflammatory drug, was used as a positive control for comparison. The experimental results revealed that both **1** (5 μM) and **2** (10 and 20 μM) exerted noticeable effects on the expression of endogenous inflammatory factor IL-1β mRNA within the cells. Specifically, Compound **1** at 5 μM significantly reduced the expression of IL-1β mRNA, resulting in an impressive inhibition rate of 31.14%. Similarly, Compound **2** at a concentration of 20 μM exhibited a significant reduction in the level of IL-1β mRNA, with an inhibition rate of 27.57% (Figure 4). These findings suggested that both **1** and **2** possessed anti-inflammatory properties. Compound **1** showed activity only at 5 μM and was inactive at higher concentrations. The cellular toxicity of **1** on LPS-stimulated C57BL/6 mouse primary bone marrow-derived macrophages at 10 and 20 μM was speculated to be the reason behind these results. Therefore, the difference in activity between compounds **1** and **2** may be attributed to their distinct configurations at C-7. In addition, anti-inflammatory activities have also been found in sesquiterpene derivatives in the published literature [26,27], proving that these types of compounds indeed have certain research value in anti-inflammatory activity. Further studies are needed to elucidate the underlying mechanisms and explore their potential in the treatment of inflammatory diseases.

During the in vitro antitumor activity screening, compounds **1**, **3**–**6**, **8**–**10**, and **14** were evaluated at a concentration of 50 μM. The results indicated that the inhibition rate on MDA-MB-435 tumor cells (human breast cancer cells) was below 50%, indicating that their IC_50_ values were greater than 50 μM.

## 3. Materials and Methods

### 3.1. General Experimental Procedures

The UV and ECD spectra was recorded on a UV-Vis spectrophotometer model 8453VU-Vis (Agilent, Beijing, China) and a chirascan circular dichroism spectrometer (Applied Photophysics, Surrey, Britain), respectively. The IR spectrum was obtained using an IR Affinity-1 spectrometer (Shimadzu, Beijing, China). HRESIMS spectra were recorded with a Bruker maXis Q-TOF mass spectrometer. The NMR spectra were recorded on a AVANCE III HD 600 MHz spectrometer (Bruker BioSpin International AG, Fällanden, Switzerland), and chemical shifts were recorded as *δ*-values. High-Performance Liquid Chromatograph (HPLC) was performed on the Agilent 1260 with a DAD detector using an ODS column (YMC-pack ODS-A, 10 × 250 mm, 5 µm). Thin-layer chromatography analysis (TLC) and column chromatography (CC) were carried out on plates precoated with silica gel GF254 (10–40 µm), over silica gel (200–300 mesh) (Qingdao Marine Chemical Factory, Qingdao, China) and Sephadex LH-20 (Amersham Biosciences, Uppsala, Sweden). Spots were detected on TLC (Qingdao Marine Chemical Factory) under 254 nm UV light. All solvents used, except for the liquid chromatography mobile phase, were of analytical grade (Tianjin Fuyu Chemical and Industry Factory, Tianjin, China). The mobile phase for liquid chromatography was of HPLC gradient grade (Shanghai Xingke High Purity Solvents Co., Ltd, Shanghai, China).

### 3.2. Fungal Source and Strain Identification

The fungal strain SCSIO 41427 was isolated from a mangrove sediment sample collected from a Gaoqiao mangrove in Lianjiang, China. The strains were stored on MB agar (malt extract 15 g, sea salt 10 g, agar 16 g, H_2_O 1 L, pH 7.4–7.8) slants in liquefied petrolatum and deposited at Key Laboratory of Tropical Marine Bio-resources and Ecology, Chinese Academy of Sciences. The strain SCSIO 41427 was designated as *Roussoella* sp., due to its ITS sequence (GenBank accession No. OR574981) homology with *Roussoella* sp. LT796863.1.

### 3.3. Fungal Cultivation and Fermentation

The fermentation of *Roussoella* sp. SCSIO 41427 was carried out using a solid-state culture medium. The preparation of the medium involved combining 180 mL of distilled water, 3 g of sea salt, and 150 g of rice in a 1000 mL conical flask. The MB seed solution was prepared by mixing 400 mL of distilled water, 8 g of sea salt, and 6 g of malt extract in a 1000 mL conical flask, with pH adjustment set to 7.4–7.8. Both media were sterilized by autoclaving at 121 °C for 30 min and allowed to cool. The strain was activated by inoculating it into MB agar medium (or PDA medium) after being stored in paraffin oil. The activation process occurred at 26 °C for 5 days (typically 5 days), after which agar sections containing the newly cultivated *Roussoella* sp. SCSIO 41427 were transferred to MB seed liquid. This mixture was then cultured in two bottles at 27 °C with agitation at 180 rpm for 48 h to obtain the seed liquid. The seed liquid was subsequently transferred to the solid rice culture medium and allowed to ferment at 26 °C for 30 days. This process was scaled up to 60 bottles, yielding 45 bottles of fermented material (6.75 kg in dry) from *Roussoella* sp. SCSIO 41427.

### 3.4. Extraction and Isolation

After the fermentation product was crushed, it was subjected to ultrasonic extraction with ethyl acetate and the resulting crude extract (109.1 g) was obtained. Silica gel (200–300 mesh) column chromatography (CC) was employed using stepwise gradient elution with petroleum ether/dichloromethane (0–100%, *v*/*v*) and dichloromethane/methanol (0–100%, *v*/*v*) to obtain six fractions (Frs. A–F). Fraction C (Fr. C, 5.5 g) was separated by ODS CC using CH_3_OH/H_2_O (10:90–100:0) gradient elution, yielding five subfractions (C1–C5). Subfraction C5 was further purified using semi-preparative HPLC with CH_3_CN/H_2_O (40:60, 0.1% HCOOH) as the eluent, resulting in the isolation of **1** (3.9 mg, *t*_R_ = 34.8 min) and **2** (3.4 mg, *t*_R_ = 36.3 min). Subfraction C4, obtained from semi-preparative HPLC with CH_3_OH/H_2_O (58:42) as the eluent, yielded **5** (17.0 mg, *t*_R_ = 21.8 min). Subfraction C3 was isolated using semi-preparative HPLC with CH_3_OH/H_2_O (55:45) as the eluent, resulting in the isolation of **9** (6.9 mg, *t*_R_ = 16.7 min). Subfraction C2 was purified using semi-preparative HPLC with CH_3_OH/H_2_O (40:60) as the eluent, yielding **6** (2.3 mg, *t*_R_ = 34.5 min) and **16** (20.8 mg, *t*_R_ = 31.3 min). Fraction E (2.3 g) was subjected to Sephadex LH-20 CC, eluting with methanol, to obtain three subfractions (E1–E3). Subfraction E3 was further isolated using semi-preparative HPLC with CH_3_CN/H_2_O (18:82) as the eluent, yielding **3** (7.7 mg, *t*_R_ = 12.0 min). Fraction A (5.0 g) was separated from Fr. A by ODS CC with CH_3_OH/H_2_O (10:90–100:0) gradient elution and was subsequently fractionated into four subfractions (A1–A4). Subfraction A2 was further purified using semi-preparative HPLC with CH_3_CN/H_2_O (55:45) as the eluent, resulting in the isolation of **4** (5.0 mg, *t*_R_ = 16.8 min). Subfraction A3 was obtained using semi-preparative HPLC with CH_3_OH/H_2_O (68:32) as the eluent, resulting in the isolation of **7** (6.5 mg, *t*_R_ = 25.8 min). Fractions D (2.3 g), B (2.5 g), and F (2.0 g) were subjected to Sephadex LH-20 CC with methanol as the eluent, leading to the separation of four subfractions (D1–D4), five subfractions (B1–B5), and four subfractions (F1–F4), respectively. Subfraction D2 was purified using semi-preparative HPLC with CH_3_OH/H_2_O (25:75) as the eluent, yielding **14** (5.2 mg, *t*_R_ = 20.3 min), D3 with CH_3_OH/H_2_O (45:55) as the eluent, yielding **8** (7.8 mg, *t*_R_ = 21.8 min) and **17** (4.5 mg, *t*_R_ = 11.0 min), D4 with CH_3_OH/H_2_O (40:60, 0.1% HCOOH) as the eluent, yielding **12** (7.0 mg, *t*_R_ = 19.5 min). Subfraction B5 was isolated using semi-preparative HPLC with CH_3_CN/H_2_O (46:54) as the eluent, leading to the isolation of **10** (2.6 mg, *t*_R_ = 18.4 min). Subfraction F2 was purified using semi-preparative HPLC with CH_3_OH/H_2_O (15:85, 0.1% HCOOH) as the eluent, resulting in the isolation of **15** (22.5 mg, *t*_R_ = 13.2 min). Subfraction F3 was purified using semi-preparative HPLC with CH_3_CN/H_2_O (20:80, 0.1% HCOOH) as the eluent, resulting in the isolation of **11** (3.9 mg, *t*_R_ = 18.8 min). Subfraction F4 was isolated using semi-preparative HPLC with CH_3_OH/H_2_O (5:95) as the eluent, leading to the isolation of **13** (2.4 mg, *t*_R_ = 13.0 min).

### 3.5. Spectroscopic Data of ***1**–**3***

Elgonene M (**1**): pale yellow oil; ${\left[\alpha \right]}_{D}^{25}$ −25.9 (*c* 0.1, CH_3_OH); ECD (0.2 mg/mL, CH_3_OH) *λ*_max_ *(∆ε*) 201 (−4.41), 220 (1.25), 262 (−4.35); UV (CH_3_OH) λ_max_ (log *ε*) 261 (0.62) nm; IR*υ*_max_: 3440, 2961, 2924, 2855, 1682, 1636, 1609, 1377, 978 cm^−1^; ^1^H and ^13^C NMR data, as shown in Table 1; HRESIMS *m*/*z* 249.1499 [M-H]^−^ (calculated for C_15_H_21_O_3_^−^, 249.1496).

Elgonene N (2): pale yellow oil; ${\left[\alpha \right]}_{D}^{25}$ −18.4 (*c* 0.1, CH_3_OH); ECD (0.2 mg/mL, CH_3_OH) *λ*_max_ (*∆ε*) 201 (−6.44), 219 (0.32), 258 (−4.91); UV (CH_3_OH) *λ*_max_ (log *ε*) 260 (0.70) nm; IR*υ*_max_: 3440, 2959, 2922, 2853, 1682, 1639, 1404, 1379, 1018, 980 cm^−1^; ^1^H and ^13^C NMR data as shown in Table 1; HRESIMS *m*/*z* 249.1498 [M-H]^−^ (calculated for C_15_H_21_O_3_^−^, 249.1496).

Methyl 5-*O*-acetyl-5-*epi*-shikimate (3): pale yellow oil; ${\left[\alpha \right]}_{D}^{25}$ −49.6 (*c* 0.1, CH_3_OH); ECD (0.2 mg/mL, CH_3_OH) *λ*_max_ (∆*ε*) 200 (32.65), 223 (−53.34), 254 (5.08); UV (CH_3_OH) *λ*_max_ (log *ε*) 218 (0.65) nm; ^1^H and ^13^C NMR data as shown in Table 1; HRESIMS *m*/*z* 231.0870 [M+H]^+^ (calculated for C_10_H_15_O_6_^+^, 231.0863) and 253.0690 [M+Na]^+^ (calculated for C_10_H_14_NaO_6_^+^, 253.0683).

### 3.6. ECD Calculation of ***1**–**3***

Conformational analyses of **1**–**3** were carried out by Spartan’14 software (v1.1.4, Wavefunction, Irvine, CA, USA) using a Molecular Merck force field. The conformers with a Boltzmann population exceeding 1% were subsequently optimized by utilizing Gaussion09 (D.01, Pittsburgh, PA, USA) at the B3LYP/6-31G (d) level in methanol using the PCM model [28]. The optimized stable conformers were chosen for further ECD calculations at the B3LYP/6-311G (d, p) level in methanol. The rotatory strengths for a total of 20 excited states were calculated. The overall ECD data were weighted by Boltzmann distribution, and the ECD curves and enantiomeric ECD curves were generated by GuassianView 6.0 software with a half-bandwidth of 0.33 eV, based on the Boltzmann-calculated contribution of each conformer after UV correction.

### 3.7. Anti-Inflammatory Assay

Femurs and tibias were harvested from 6–8 week old C57BL/6 mice, rinsed thrice with 2% antibiotic-containing PBS, and both ends of the bones were removed. Bone marrow cavities were flushed with PBS, and the cell suspension was passed through a 70 μm cell strainer into a centrifuge tube. The suspension was centrifuged at 500× *g* for 5 min at 4 °C, the supernatant was discarded, and red blood cells were lysed by adding red blood cell lysis buffer and incubating for 10 min. Following another centrifugation at 500× *g* for 5 min at 4 °C, the supernatant was discarded, and cells were resuspended in α-MEM containing 10% FBS. The cells were seeded in culture dishes and incubated in a 37 °C, 5% CO_2_ incubator for 2 h. The medium was then collected in centrifuge tubes, centrifuged at 500× *g* for 5 min at 4 °C, and cells were resuspended in α-MEM containing 50 ng/mL M-CSF. Cells were seeded at a density of 2.5 × 10^6^ cells/mL in a 24-well plate and cultured for 5 days for subsequent experiments. The cells were divided into a blank group, a model group, treatment groups with compounds **1** and **2** (at concentrations of 1 μM, 5 μM, 10 μM, and 20 μM), and a positive control group with dexamethasone (20 μM). Except for the blank group, all other groups were co-incubated with 100 ng/mL lipopolysaccharide (LPS) for 6 h. After 6 h, the IL-1β mRNA expression was measured. The culture medium was discarded, and the cells were washed twice with PBS. RNA was extracted using the Trizol method, and its concentration was determined with NanoDrop. RNA was reverse transcribed into cDNA using the EnzyArtisan reverse transcription kit. A 10 μL qPCR reaction was prepared according to the SYBR qPCR kit (EnzyArtisan) instructions and run on a ROCHE qPCR LightCycler96 instrument following the recommended protocol. The housekeeping gene GAPDH was used as an internal control to normalize the CT values, using the 2^−∆∆Ct^ formula. The results were shown as mean ± SD from three independent experiments.

### 3.8. Cytotoxicity Bioassay

The cytotoxicity of **1**, **3**–**6**, **8**–**10**, and **14** against MDA-MB-435 (human breast cancer cells) was determined by assessing cell viability through the 3-(4,5)-dimethylthiahiazo (-z-yl)-3,5-di-phenytetrazoliumromide (MTT) assay. Briefly, cells were seeded at a density of 5 × 10^3^ cells per well in a 96-well plate and left to incubate overnight, followed by treatment with the compounds for the required duration. The optical density at 570 nm (OD_570_) was measured using a Hybrid Multi-Mode Reader (Synergy H1, BioTek, Santa Clara, CA, USA). The experiment was independently repeated three times.

## 4. Conclusions

In summary, seventeen compounds, including two new sesquiterpenoid derivatives (**1**–**2**) and a new shikimic acid metabolite (**3**), were isolated from the mangrove-sediment fungus *Roussoella* sp. SCSIO 41427. The absolute configurations of the new compounds were confirmed by ECD calculations and NMR data analysis. During the screening for activity against MDA-MB-435 tumor cells, the compounds **1**, **3**–**6**, **8**–**10**, and **14** exhibited an inhibition rate below 50%, indicating a lack of significant inhibitory activity. Moreover, the newly discovered sesquiterpenoid derivatives **1** and **2** exhibited inhibitory effects on the expression of the inflammatory factor IL-1β mRNA, suggesting their potential as anti-inflammatory agents worthy of further investigation.

## Figures and Tables

**Table 1:** The NMR data of **1**–**3** (600 and 150 MHz, *δ* in ppm, CDCl_3_).

Pos.	1		2		3	
	*δ*_C_, Type	*δ*_H_, (*J* in Hz)	*δ*_C_, Type	*δ*_H_, (*J* in Hz)	*δ*_C_, Type	*δ*_H_, (*J* in Hz)
1	173.5, C		173.1, C		129.2, C	
2	126.1, C		126.0, C		140.5, CH	6.79, brs
3	140.1, CH	7.29, d, (11.4)	140.1, CH	7.30, d, (11.4)	69.2, CH	4.42, m
4	123.5, CH	6.59, dd, (15.1, 11.4)	123.3, CH	6.61, dd, (15.2, 11.4)	69.7, CH	4.08, m
5	148.0, CH	6.17, d, (15.1)	148.8, CH	6.17, d, (15.2)	72.4, CH	5.05, m
6	75.3, C		75.5, C		26.5, CH_2_	2.63, m
7	44.3, CH	1.63, m	44.2, CH	1.63, m	168.1, C	
8	26.9, CH_2_	1.81, m; 2.03, m	25.8, CH_2_	1.81, m; 2.03, m	52.4, CH_3_	3.76, s
9	120.3, CH	5.36, m	120.4, CH	5.36, m	172.3, C	
10	134.2, C		134.2, C		21.0, CH_3_	2.11, s
11	30.8, CH_2_	1.99, m	30.9, CH_2_	1.99, m		
12	23.6, CH_2_	1.24, m; 1.90, m	24.2, CH_2_	1.24, m; 1.90, m		
13	23.5, CH_3_	1.64, s	23.5, CH_3_	1.64, s		
14	26.1, CH_3_	1.33, s	26.3, CH_3_	1.32, s		
15	12.6, CH_3_	1.96, s	12.6, CH_3_	1.97, s		

## Data Availability

The data presented in this study are available on request from the corresponding author.

## Supplementary Materials

The following supporting information can be downloaded at: https://www.mdpi.com/article/10.3390/md22030103/s1, Figures S1–S32: NMR, HRESIMS, UV, IR, CD and HPLC spectra of compounds **1**–**3**; Table S1 and S2: Energies of **1**–**3** at B3LYP/6–311g (d, p) level; The physicochemical and spectroscopic data of compounds **4**–**17**.
