# Supplementary material for: Two New Sesquiterpenoids and a New Shikimic Acid Metabolite from Mangrove Sediment-Derived Fungus Roussoella sp. SCSIO 41427
Source: Mar Drugs. 2024 Feb 23;22(3):103. doi: 10.3390/md22030103 (PMC10971368; doi:10.3390/md22030103)
Supplement: Supplementary file 1 [file marinedrugs-22-00103-s001.zip › marinedrugs-2844093-supplementary.pdf]

Supporting Information

Two New Sesquiterpenoids and a New Shikimic Acid Metabolite from Mangrove Sediment-Derived Fungus *Roussoella* sp. SCSIO 41427

Zimin Xiao [1,†], Jian Cai [2,†], Ting Chen [3], Yilin Wang [3], Yixin Chen [1], Yongyan Zhu [1], Chunmei Chen [2], Bin Yang [2], Xuefeng Zhou [2] and Huaming Tao [1,*]

[1] Guangdong Provincial Key Laboratory of Chinese Medicine Pharmaceutics, School of Traditional Chinese
Medicine, Southern Medical University, Guangzhou 510515, China; 15917491112@163.com (Z.X.); 13676126834@163.com (Y.C.); yongyanzhu0521@163.com (Y.Z.)

[2] CAS Key Laboratory of Tropical Marine Bio-Resources and Ecology, Guangdong Key Laboratory of Marine
Materia Medica, South China Sea Institute of Oceanology, Chinese Academy of Sciences, Guangzhou 510301, China; caijian19@mails.ucas.ac.cn (J.C.); chenchunmei18@mails.ucas.ac.cn (C.C.); yangbin@scsio.ac.cn (B.Y.); xfzhou@scsio.ac.cn (X.Z.)

[3] School of Stomatology, Southern Medical University, Guangzhou 510515, China; chent@smu.edu.cn (T.C.); FOURCWATER@outlook.com (Y.W.)

* Correspondence: taohm@smu.edu.cn; Tel.: +86-020-61648770

† These authors contributed equally to this work.

Table of Contents

The physicochemical data of the known compounds **4-17**.

Figure S1. ^{1}H NMR spectrum of elgonene M (**1**) in $CDCl_3$

Figure S2. ^{13}C NMR spectrum of elgonene M (**1**) in $CDCl_3$

Figure S3. DEPT135 spectrum of elgonene M (**1**) in $CDCl_3$

Figure S4. HSQC spectrum of elgonene M (**1**) in $CDCl_3$

Figure S5. HMBC spectrum of elgonene M (**1**) in $CDCl_3$

Figure S6. ^{1}H-^{1}H COSY spectrum of elgonene M (**1**) in $CDCl_3$

Figure S7. NOESY spectrum of elgonene M (**1**) in $CDCl_3$

Figure S8. HRESIMS spectrum of elgonene M (**1**)

Figure S9. IR spectrum of elgonene M (**1**)

Figure S10. UV spectrum of elgonene M (**1**) in CH_3OH

Figure S11. ECD spectrum of elgonene M (**1**) in CH_3OH

Figure S12. ^{1}H NMR spectrum of elgonene N (**2**) in $CDCl_3$

Figure S13. ^{13}C NMR spectrum of elgonene N (**2**) in $CDCl_3$

Figure S14. DEPT135 spectrum of elgonene N (**2**) in $CDCl_3$

Figure S15. HSQC spectrum of elgonene N (**2**) in $CDCl_3$

Figure S16. HMBC spectrum of elgonene N (**2**) in $CDCl_3$

Figure S17. ^{1}H-^{1}H COSY spectrum of elgonene N (**2**) in $CDCl_3$

Figure S18. NOESY spectrum of elgonene N (**2**) in $CDCl_3$

Figure S19. HRESIMS spectrum of elgonene N (**2**)

Figure S20. IR spectrum of elgonene N (**2**)

Figure S21. UV spectrum of elgonene N (**2**) in CH_3OH

Figure S22. ECD spectrum of elgonene N (**2**) in CH_3OH

Figure S23. ^1H NMR spectrum of compound **3** in $CDCl_3$

Figure S24. ^{13}C NMR spectrum of compound **3** in $CDCl_3$

Figure S25. HSQC spectrum of compound **3** in CD_3OD

Figure S26. HMBC spectrum of compound **3** in CD_3OD

Figure S27. ^1H-^1H COSY spectrum of compound **3** in CD_3OD

Figure S28. NOESY spectrum of compound **3** in CD_3OD

Figure S29. HRESIMS spectrum of compound **3**

Figure S30. UV spectrum of compound **3** in CH_3OH

Figure S31. ECD spectrum of compound **3** in CH_3OH

Figure S32. HPLC spectrum of compounds **1** and **2**

Table S1. Energies of **1** and **2** at B3LYP/6–311g (d, p) level.

Table S2. Energies of **3** at B3LYP/6–311g (d, p) level.

Compound **4**: White powder, HRESIMS *m/z* 229.0469 $[M+Na]^+$ (calculated for $C_{11}H_{10}NaO_4^+$, 229.0471); ^{1}H NMR (600 MHz, $CDCl_3$) δ 11.10 (1H, chelating phenol), 6.45 (1H, d, J = 2.3 Hz, H-5), 6.29 (1H, d, J = 2.3 Hz, H-7), 6.17 (1H, d, J = 1.3 Hz, H-4), 3.86 (3H, s, OMe), 2.25 (3H, d, J = 1.0 Hz, H-9); ^{13}C NMR (150 MHz, $CDCl_3$) δ 166.9 (C-1), 166.5 (C-6), 163.8 (C-8), 154.4 (C-3), 139.6 (C-4a), 104.7 (C-4), 101.1 (C-5), 100.3 (C-7), 99.9 (C-8a), 55.8 (C-OMe), 19.6 (C-9).

Compound **5**: Pale yellow powder, HRESIMS *m/z* 273.0739 $[M+Na]^+$ (calculated for $C_{13}H_{14}NaO_5^+$, 273.0733); ^{1}H NMR (600 MHz, CD_3OD) δ 6.48 – 6.42 (3H, m, H-4, H-7, H-5), 4.21 – 4.12 (1H, m, H-12), 3.88 (3H, OMe), 2.66 – 2.56 (2H, m, H-11), 1.27 (3H, d, J = 6.3 Hz, H-13). ^{13}C NMR (150 MHz, CD_3OD) δ 168.5 (C-1), 167.8 (C-8), 164.6 (C-6), 156.5 (C-3), 141.1 (C-10), 107.1 (C-4), 102.1 (C-7), 101.5 (C-5), 100.9 (C-9), 66.2 (C-12), 56.3 (C-OMe), 43.8 (C-11), 23.4 (C-13).

Compound **6**: Red-brown gel-like substance, HRESIMS *m/z* 275.0895 $[M+Na]^+$ (calculated for $C_{13}H_{16}NaO_5^+$, 275.0890); ^{1}H NMR (600 MHz, $CDCl_3$) δ 7.85 (1H, -OH), 6.43 (1H, s, H-7), 4.21 (1H, q, J = 6.5 Hz, H-3), 3.88 (3H, s, H-12), 2.11 (3H, s, H-11), 1.80 (3H, s, H-10), 0.93 (3H, d, J = 6.4 Hz, H-9).^{13}C NMR (150 MHz, $CDCl_3$) δ 171.7 (C-1), 165.5 (C-6), 156.7 (C-8), 150.0 (C-4a), 112.4 (C-5), 103.1 (C-8a), 98.4 (C-7), 92.1 (C-4), 71.1 (C-3), 56.4 (C-12), 21.4 (C-10), 18.0 (C-9), 11.4 (C-11).

Compound **7**: White powder, HRESIMS *m/z* 257.0789 $[M+Na]^+$ (calculated for $C_{13}H_{14}NaO_4^+$, 257.0784); ^{1}H NMR (600 MHz, $CDCl_3$) δ 11.02 (1H, s, -OH), 6.38 (1H, s, H-7), 4.79 (1H, d, J = 1.9 Hz, H-9b), 4.56 (1H, d, J = 1.9 Hz, H-9a), 3.86 (3H, s, H-12), 3.81 (1H, q, J = 7.1 Hz, H-4), 2.07 (3H, s, H-11), 1.37 (3H, d, J = 7.1 Hz, H-10).^{13}C NMR (150 MHz, $CDCl_3$) δ 167.1 (C-1), 164.9 (C-6), 163.1 (C-8), 157.2 (C-3), 142.2 (C-4a), 114.1 (C-5), 98.7 (C-8a), 97.6 (C-7), 96.0 (C-8), 56.0 (C-12), 35.0 (C-4), 22.4 (C-10), 10.1 (C-11).

Compound **8**: Red-brown crystals, ^{1}H NMR (600 MHz, CD_3OD) δ 6.36 (1H, s, H-4), 6.31 (1H, d, J = 2.1 Hz, H-7), 6.30 (1H, d, J = 2.2 Hz, H-5), 4.18 – 4.12 (1H, m, H-2'), 2.64 – 2.54 (2H, m, H-1'), 1.25 (3H, d, J = 6.2 Hz, H-3'). ^{13}C NMR (150 MHz, CD_3OD) δ 167.9 (C-1), 167.3 (C-8), 164.9 (C-6), 156.2 (C-3), 141.3 (C-10), 107.0 (C-4), 103.7 (C-7), 102.6 (C-5), 99.8 (C-9), 66.2 (C-2'), 43.8 (C-1'), 23.3 (C-3').

Compound **9**: Brown powder, ^{1}H NMR (600 MHz, CD_3OD) δ 6.29 (2H, t, J = 2.3 Hz, H-4, H-5), 6.26 (1H, d, J = 2.0 Hz, H-7), 2.22 (3H, s, H-9). ^{13}C NMR (150 MHz, CD_3OD) δ 167.8 (C-6), 167.4 (C-1), 164.9 (C-8), 155.5 (C-3), 141.5 (C-4a), 105.5 (C-5), 103.4 (C-7), 102.5 (C-4), 99.5 (C-8a), 19.2 (C-9).

Compound **10**: White powder, ^{1}H NMR (600 MHz, CD_3OD) δ 6.52 (1H, s, H-7), 5.09 (1H, d, J = 1.7 Hz, H_a-9), 4.88 (1H, d, J = 1.7 Hz, H_b-9), 3.89 (3H, s, H-12), 2.40 (3H, s, H-11), 1.66 (3H, s, H-10). ^{13}C NMR (CD_3OD, 150 MHz) δ 168.1 (C-1), 167.3 (C-6), 164.4 (C-8), 162.9 (C-3), 144.8 (C-4a), 117.7 (C-5), 99.3 (C-7), 99.1 (C-8a), 95.4 (C-9), 72.5 (C-4), 56.6 (C-12), 29.5 (C-10), 12.2 (C-11).

Compound **11**: Colorless gel-like substance, $[\alpha]_D^{25}$ +35.2 (c 0.1, CH_3OH); HRESIMS *m/z* 230.0787 $[M+Na]^+$ (calculated for $C_{11}H_{13}NNaO_3^+$, 230.0788); ^{1}H NMR (600 MHz, CD_3OD) δ 7.29 – 7.25 (2H, m), 7.24 – 7.18 (3H, m), 4.64 (1H, dd, J = 9.1, 5.0 Hz, H-2), 3.20 (1H, dd, J = 13.9, 5.0 Hz, H-3b), 2.94 (1H, dd, J = 13.9, 9.0 Hz, H-3a), 1.90 (3H, s, H-12). ^{13}C NMR (150 MHz, CD_3OD) δ 175.1 (C-1), 173.1 (C-11), 138.6 (C-4), 130.2 (C-6, C-8), 129.4 (C-5, C-9), 127.8 (C-7), 55.3 (C-2), 38.5 (C-3), 22.3 (C-12).

Compound **12**: Pale yellow crystals, ^{1}H NMR (600 MHz,CD_3OD) δ 8.06 (1H, d, J = 8.1 Hz, H-4), 7.93 (1H, s, H-2), 7.46 – 7.39 (1H, m, H-7), 7.21 – 7.11 (2H, m, H-5, H-6). ^{13}C NMR (150 MHz, CD_3OD) δ 169.5 (C-1), 138.2 (C-7a), 133.3 (C-2), 127.6 (C-3), 123.6 (C-5), 122.3 (C-4), 122.1 (C-6), 112.9 (C-7), 109.0 (C-3a).

Compound **13**: White powder, ^{1}H NMR (600 MHz, DMSO-d_6) δ 10.93 (2H, s, -OH), 7.39 (1H, d, J = 7.6 Hz, H-6), 5.44 (1H, d, J = 7.6 Hz, H-5). ^{13}C NMR (150 MHz, DMSO-d_6) δ 164.3 (C-4), 151.5 (C-2), 142.3 (C-6), 100.2 (C-5).

Compound **14**: Pale brown oil, ^{1}H NMR (600 MHz, DMSO-d_6) δ 7.57 (1H, t, J = 7.9 Hz, H-7), 7.16 (1H, d, J = 7.9 Hz, H-6), 6.85 (1H, d, J = 8.3 Hz, H-8), 4.84 (1H, dt, J = 11.0, 4.5 Hz, H-4), 4.40 (1H, dd, J = 13.4, 4.6 Hz, H-3), 2.45 (1H, dt, J = 11.5, 4.8 Hz, H-2b), 1.90 (1H, dt, J = 13.4, 11.6 Hz, H-2a). ^{13}C NMR (150 MHz, DMSO-d_6) δ 206.0 (C-1), 161.4 (C-9), 150.1 (C-5), 136.6 (C-7), 116.6 (C-6), 115.5 (C-8), 113.9 (C-10), 70.4 (C-4), 65.2 (C-3), 42.2 (C-2).

Compound **15**: Colorless crystals, ^{1}H NMR (600 MHz, CD_3OD) δ 6.67 (1H, tt, J = 2.6, 1.2 Hz, H-2), 4.32 (1H, tt, J = 3.9, 2.2 Hz, H-4), 3.95 (1H, dt, J = 3.7, 1.7 Hz, H-3), 3.83 (1H, ddd, J = 9.5, 5.9, 1.8 Hz, H-5), 3.74 (3H, -OMe), 2.53 (1H, ddt, J = 17.3, 6.0, 1.6 Hz, H-6b), 2.38 (1H, dddd, J = 17.2, 9.5, 3.5, 2.6 Hz, H-6a).^{13}C NMR (150 MHz, CD_3OD) δ 168.5 (C-7), 140.2 (C-2), 129.8 (C-1), 72.3 (C-4), 69.7 (C-5), 69.4 (C-3), 52.4 (C-OMe), 29.7 (C-6).

Compound **16**: Brown oil, ^{1}H NMR (600 MHz, $CDCl_3$) δ 7.07 (2H, d, J = 8.4 Hz, H-3', H-5'), 6.77 (2H, d, J = 8.5 Hz, H-2', H-6'), 5.66 (1H, s, -OH), 4.24 (2H, t, J = 7.1 Hz, H-3), 2.86 (2H, t, J = 7.1 Hz, H-2), 2.05 (3H, s, H-7'). ^{13}C NMR (150 MHz, $CDCl_3$) δ 171.8 (C-1), 154.6 (C-1'), 130.1 (C-3', C-5'), 129.7 (C-4'), 115.5 (C-2', C-6'), 65.5 (C-3), 34.3 (C-2), 21.1 (C-7').

Compound **17**: Brown powder, ^{1}H NMR (600 MHz, CD_3OD) δ 7.42 (2H, d, J = 7.3 Hz, H-2, H-6), 6.80 (1H, d, J = 8.5 Hz, H-5), 3.84 (s, 3H). ^{13}C NMR (150 MHz, CD_3OD) δ 168.9

(-COOH), 151.7 (C-3), 146.2 (C-4), 123.6 (C-6), 122.6 (C-1), 117.4 (C-5), 115.9 (C-2), 52.2 (C-OMe).

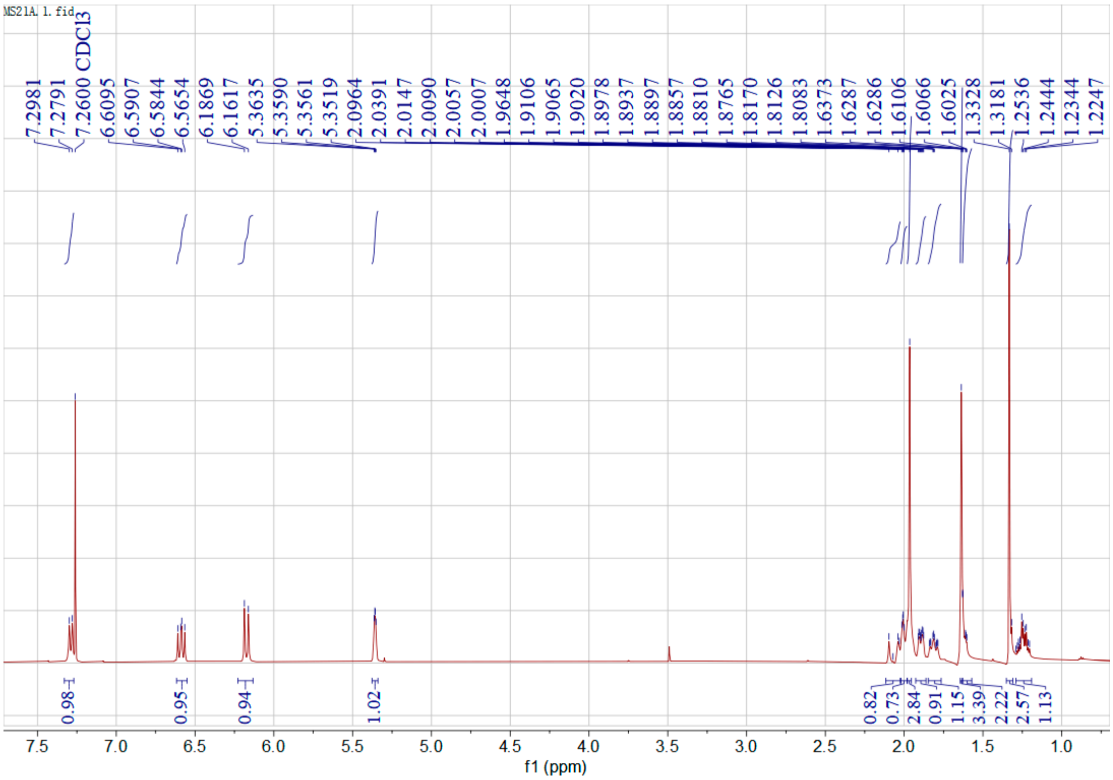

Figure S1. ^{1}H NMR spectrum of elgonene M (**1**) in $CDCl_3$

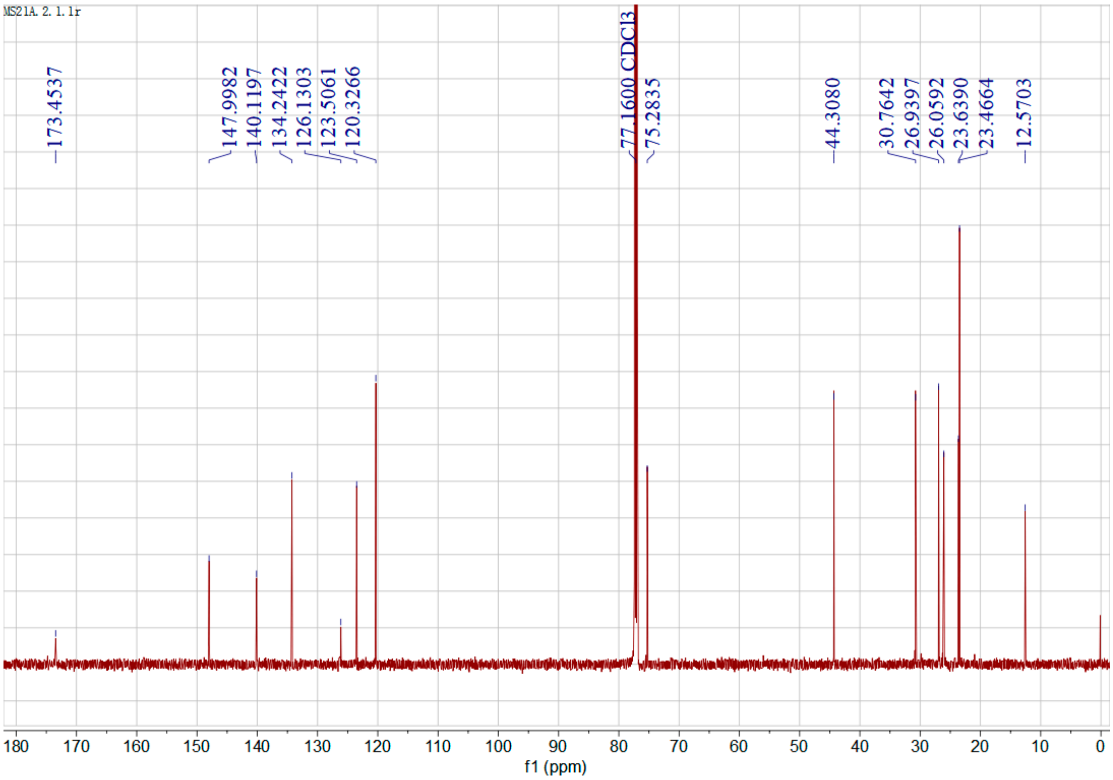

Figure S2. ^{13}C NMR spectrum of elgonene M (**1**) in $CDCl_3$

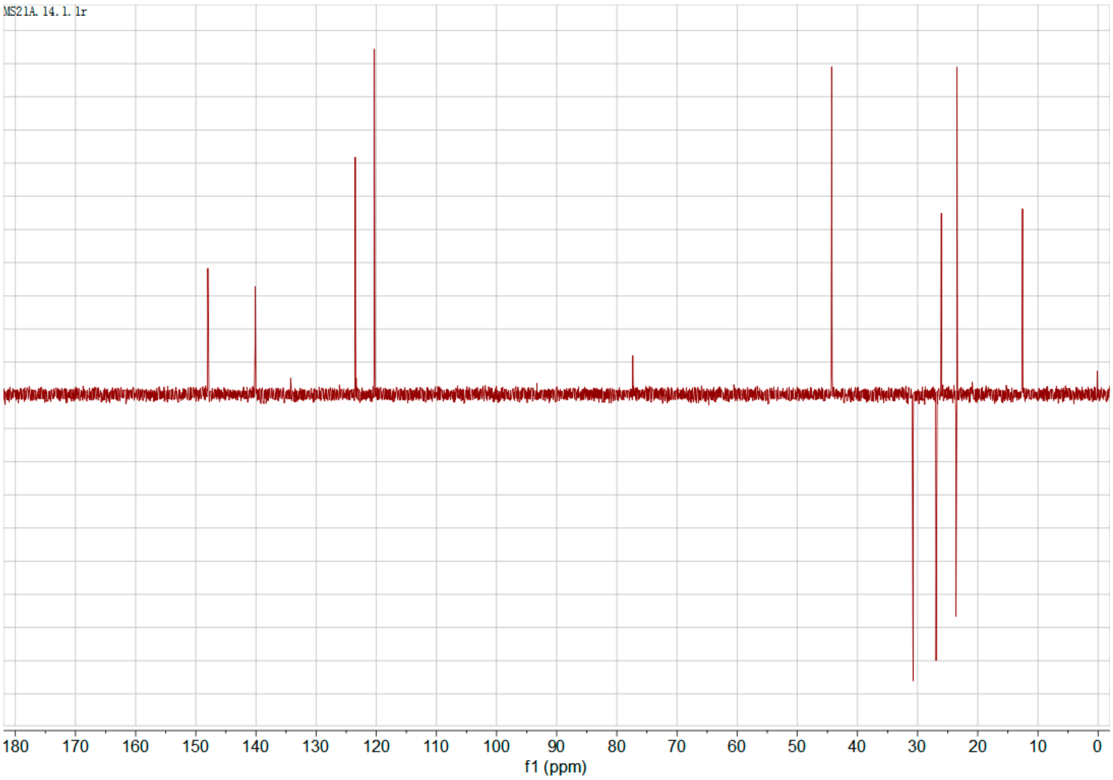

Figure S3. DEPT135 spectrum of elgonene M (**1**) in $CDCl_3$

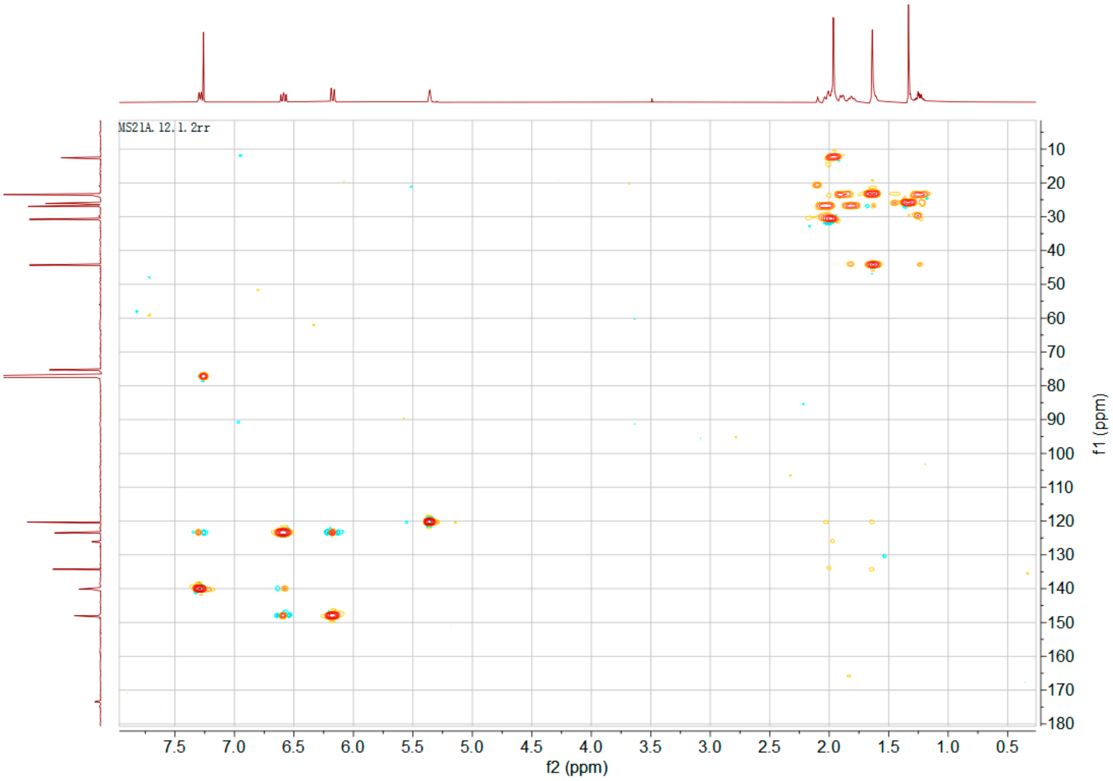

Figure S4. HSQC spectrum of elgonene M (**1**) in $CDCl_3$

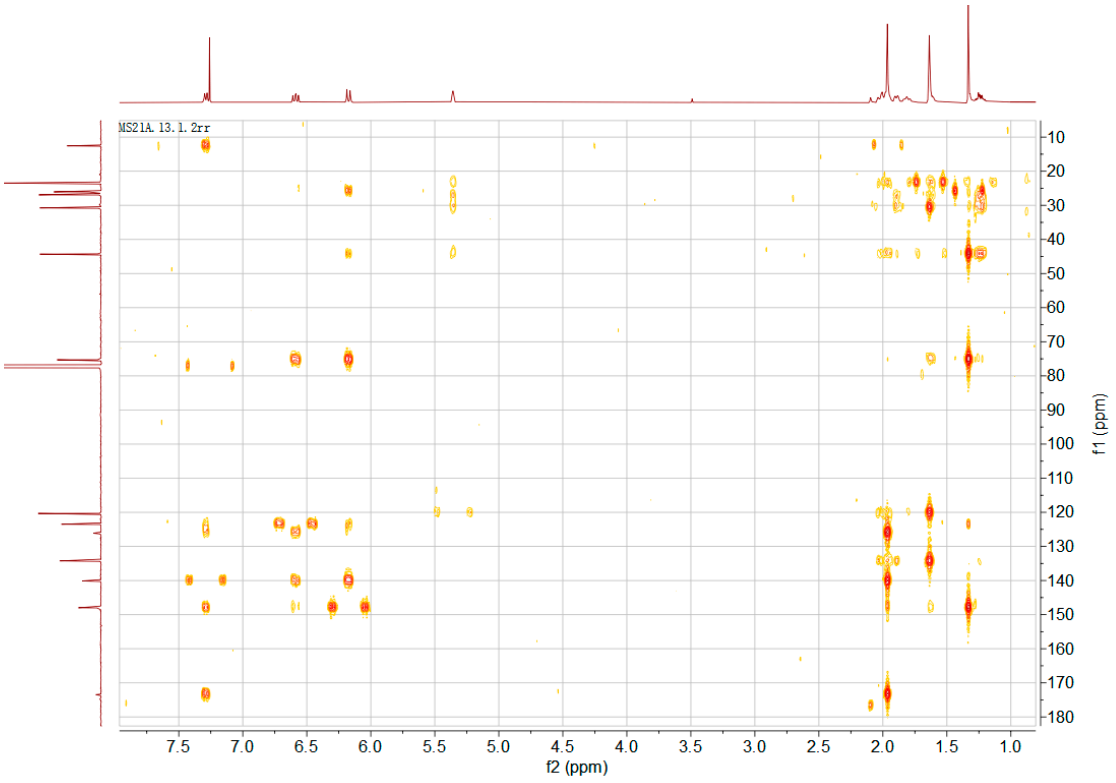

Figure S5. HMBC spectrum of elgonene M (**1**) in $CDCl_3$

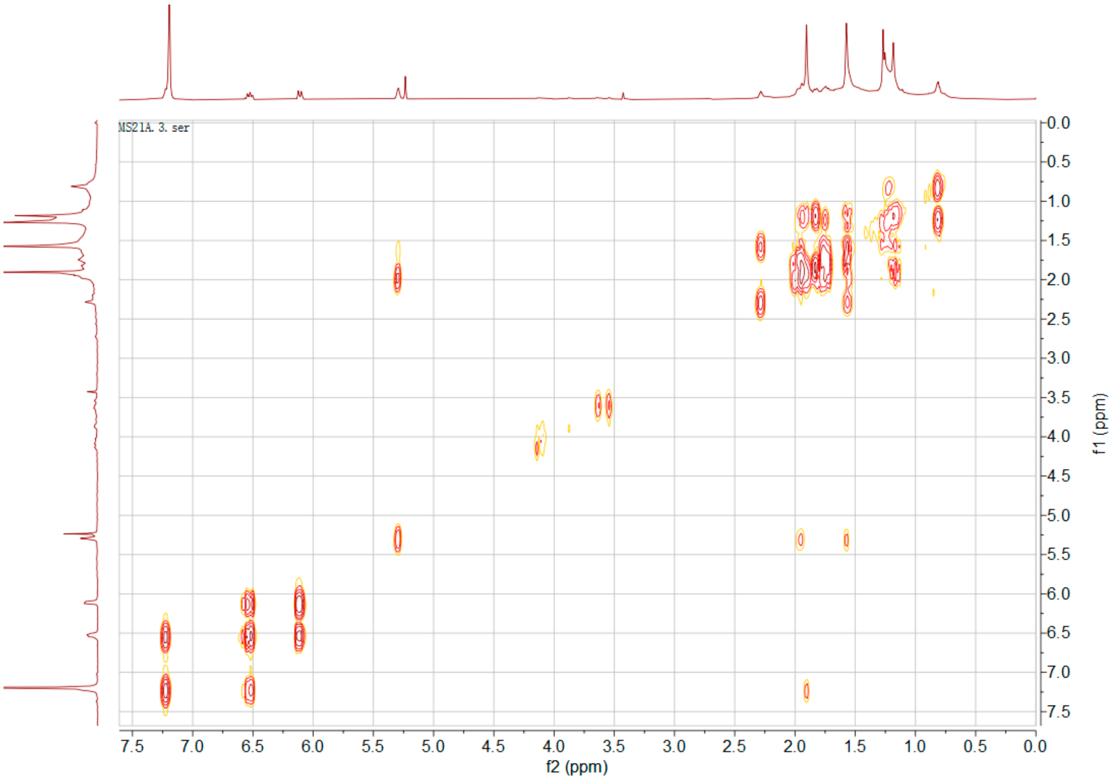

Figure S6. ^{1}H-^{1}H COSY spectrum of elgonene M (**1**) in $CDCl_3$

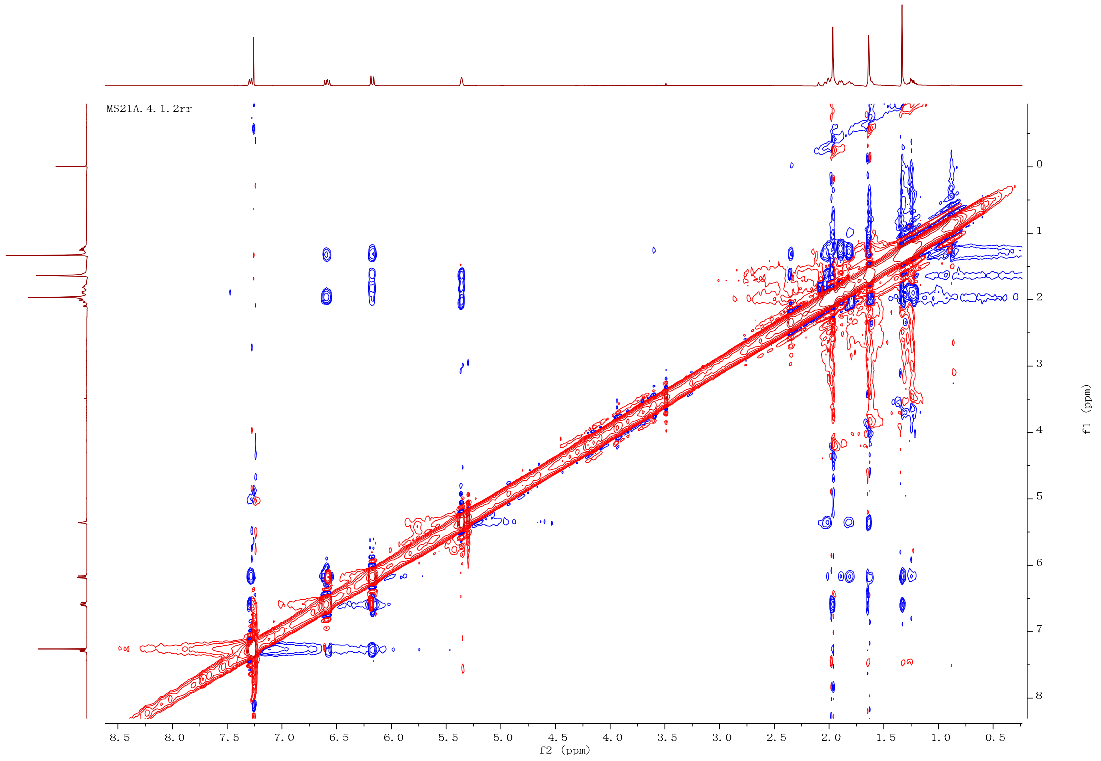

Figure S7. NOESY spectrum of elgonene M (**1**) in $CDCl_3$

Mass Spectrum SmartFormula Report

Analysis Info

Analysis Name	D:\Data\MS\data\202212\caijian_MS21A_neg_42_01_14139.d	Acquisition Date	12/13/2022 10:59:08 AM
Method	LC_Direct Infusion_neg_70-500mz.m	Operator	SCSIO
Sample Name	caijian_MS21A_neg	Instrument	maXis 255552.00029
Comment			

Acquisition Parameter

Source Type	ESI	Ion Polarity	Negative	Set Nebulizer	0.4 Bar
Focus	Active	Set Capillary	4200 V	Set Dry Heater	180 °C
Scan Begin	100 m/z	Set End Plate Offset	-500 V	Set Dry Gas	4.0 l/min
Scan End	1500 m/z	Set Charging Voltage	0 V	Set Divert Valve	Waste
		Set Corona	0 nA	Set APCI Heater	0 °C

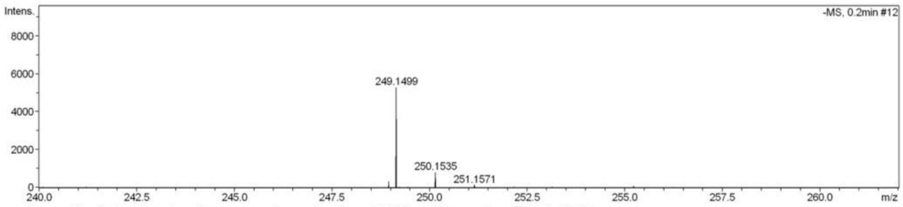

Meas. m/z	#	Ion Formula	Score	m/z	err [ppm]	err [mDa]	mSigma	rdb	e⁻ Conf	N-Rule
249.1499	1	C15H21O3	100.00	249.1496	1.2	0.3	9.3	5.5	even	ok
499.3072	1	C30H43O6	100.00	499.3065	1.3	0.6	18.3	9.5	even	ok

caijian_MS21A_neg_42_01_14139.d
Bruker Compass DataAnalysis 4.1 printed: 12/13/2022 11:04:09 AM by: SCSIO Page 1 of 1

Figure S8. HRESIMS spectrum of elgonene M (**1**)

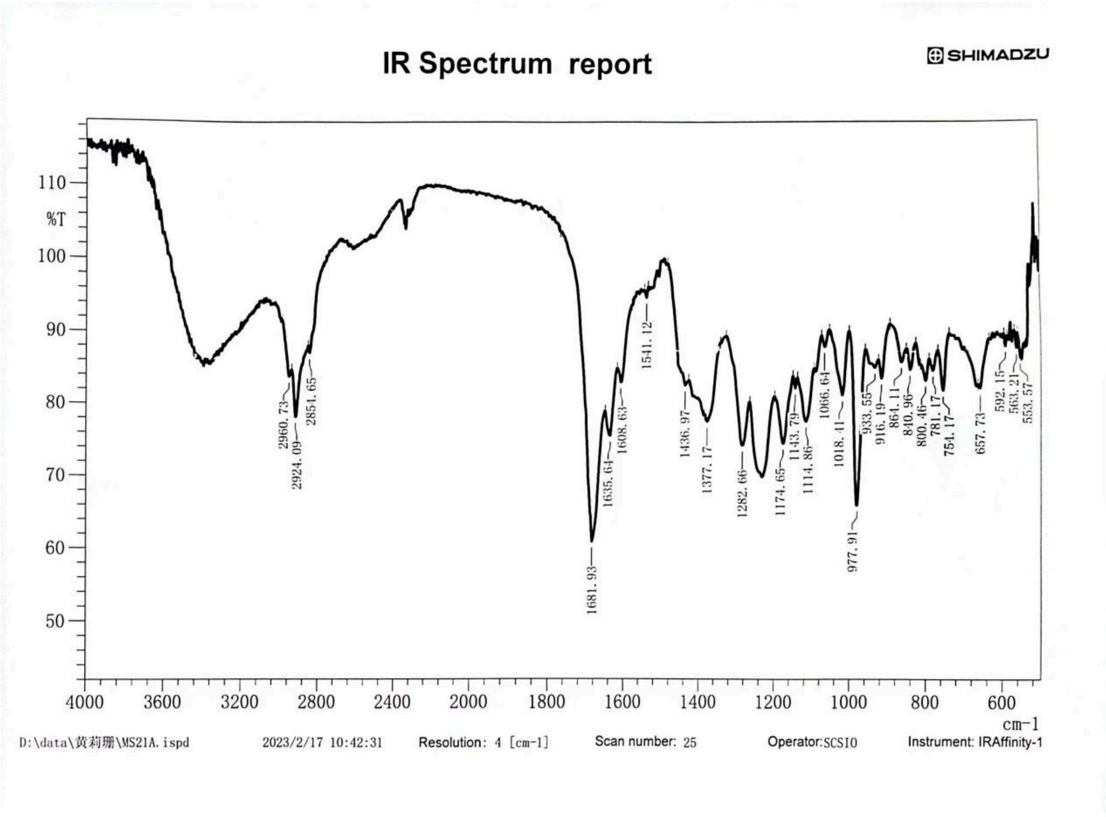

Figure S9. IR spectrum of elgonene M (**1**)

Fixed Wavelength Report   Date 2007-9-2 Time 21:17:57   Page 1 of 1

Method file : <untitled>
Information : Default Method
Data File : <untitled>

Overlaid Spectra:

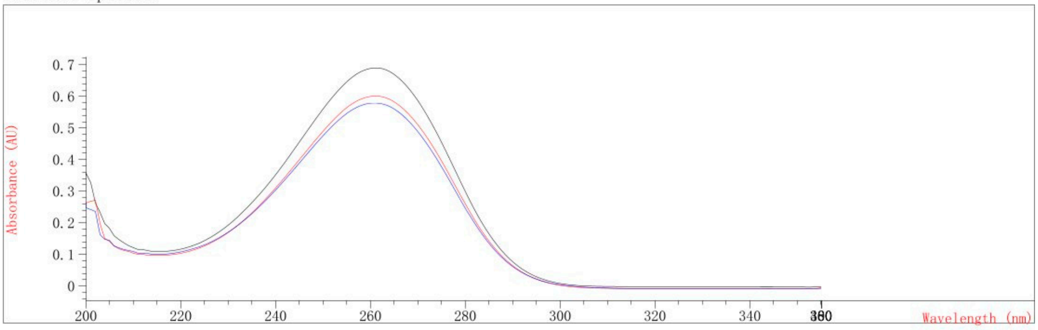

#	Name	Abs<261nm>	#	Name	Abs<261nm>	#	Name	Abs<261nm>
1		0.68986	2		0.60085	3		0.57893

Report generated by : 1   Signature: ....

*** End Fixed Wavelength Report ***

Figure S10. UV spectrum of elgonene M (**1**) in MeOH

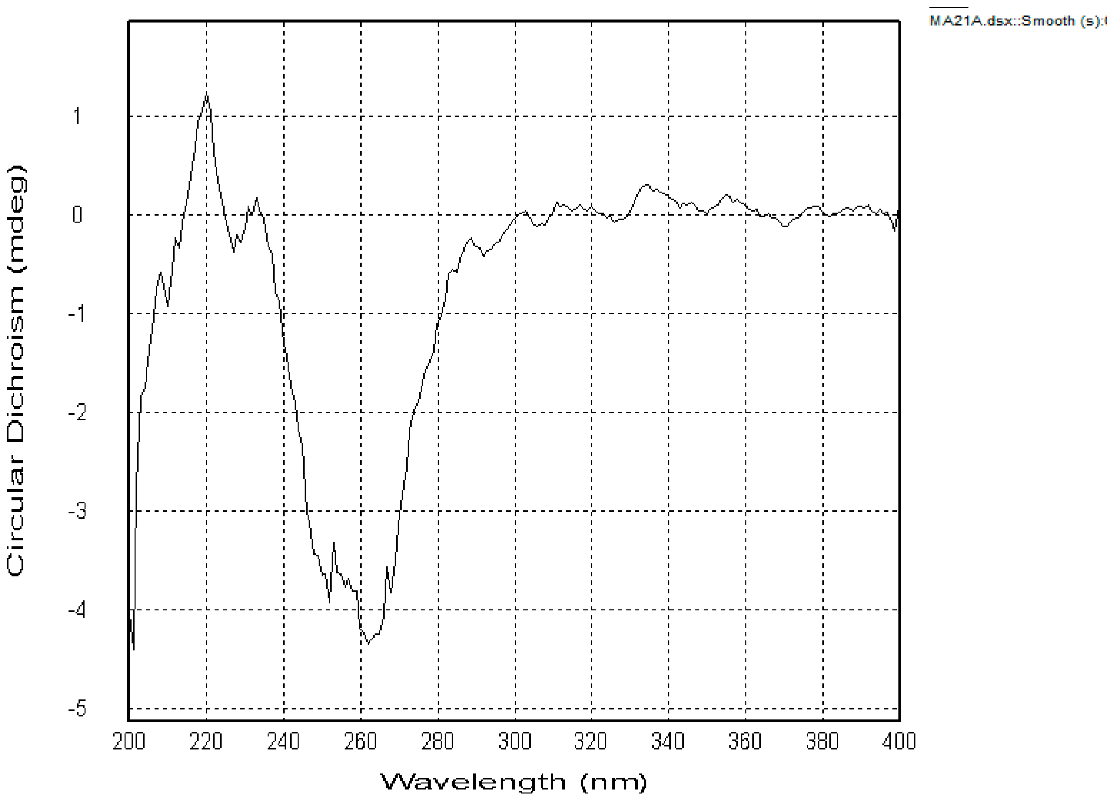

Figure S11. ECD spectrum of elgonene M (**1**) in MeOH

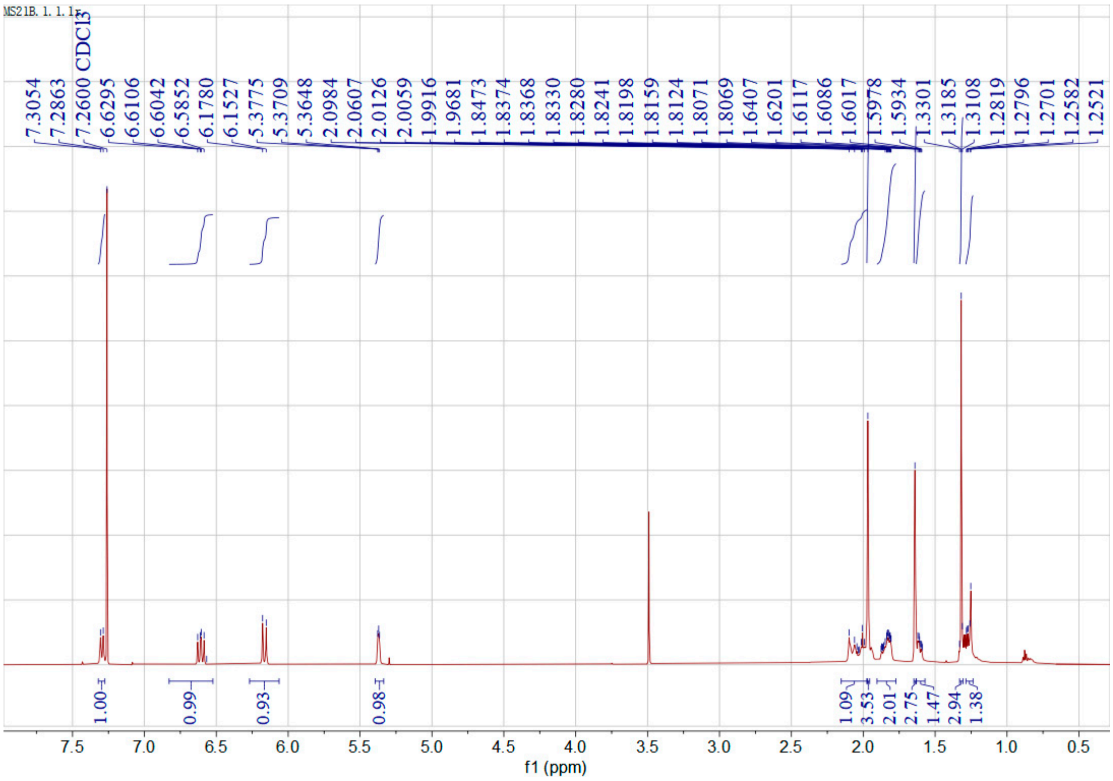

Figure S12. ^{1}H NMR spectrum of elgonene N (**2**) in $CDCl_3$

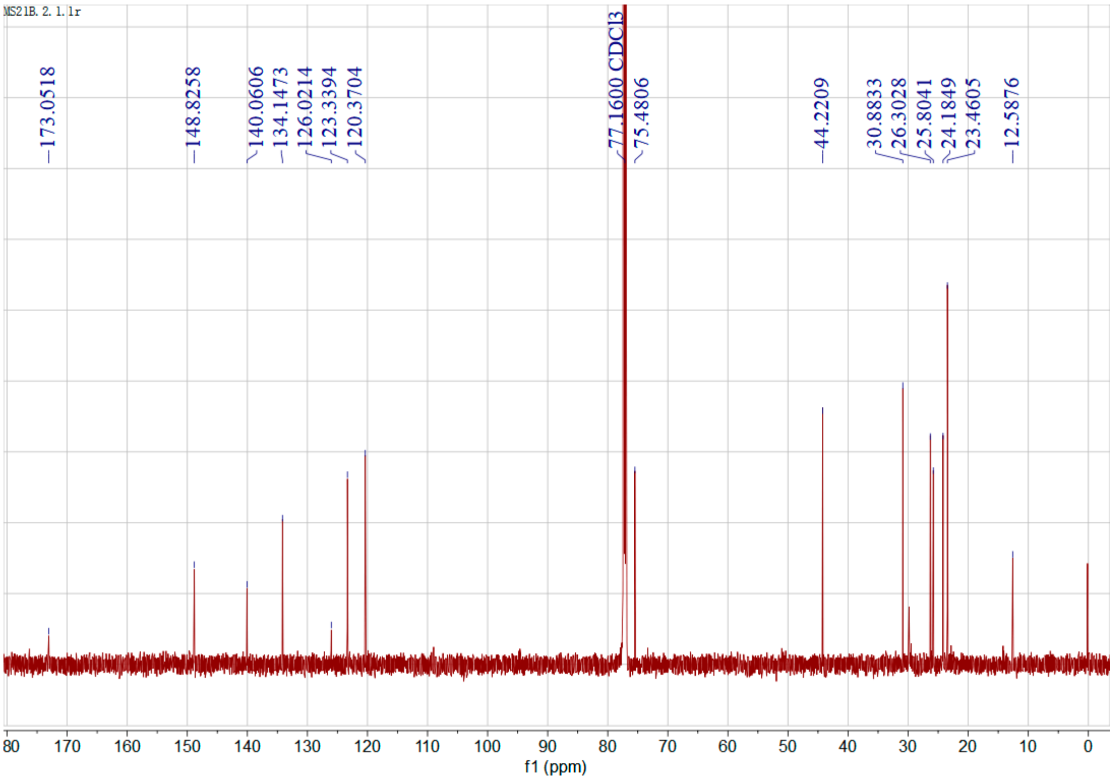

Figure S13. ^{13}C NMR spectrum of elgonene N (**2**) in $CDCl_3$

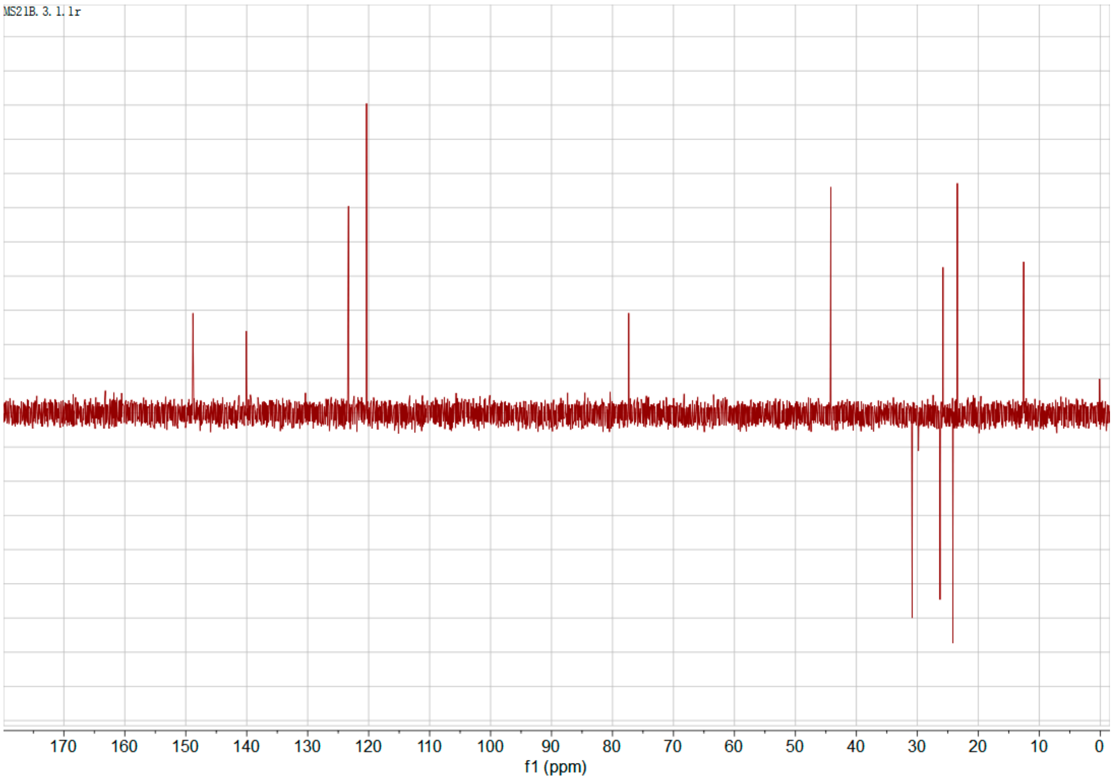

Figure S14. DEPT135 spectrum of elgonene N (**2**) in $CDCl_3$

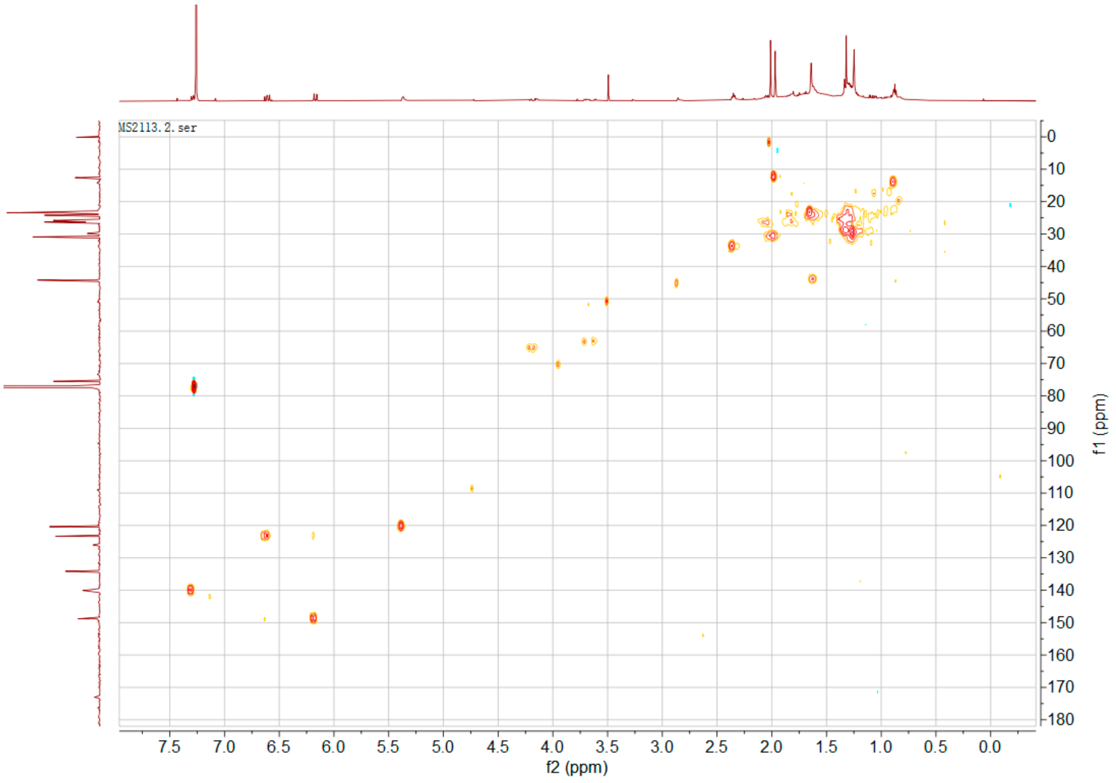

Figure S15. HSQC spectrum of elgonene N (**2**) in $CDCl_3$

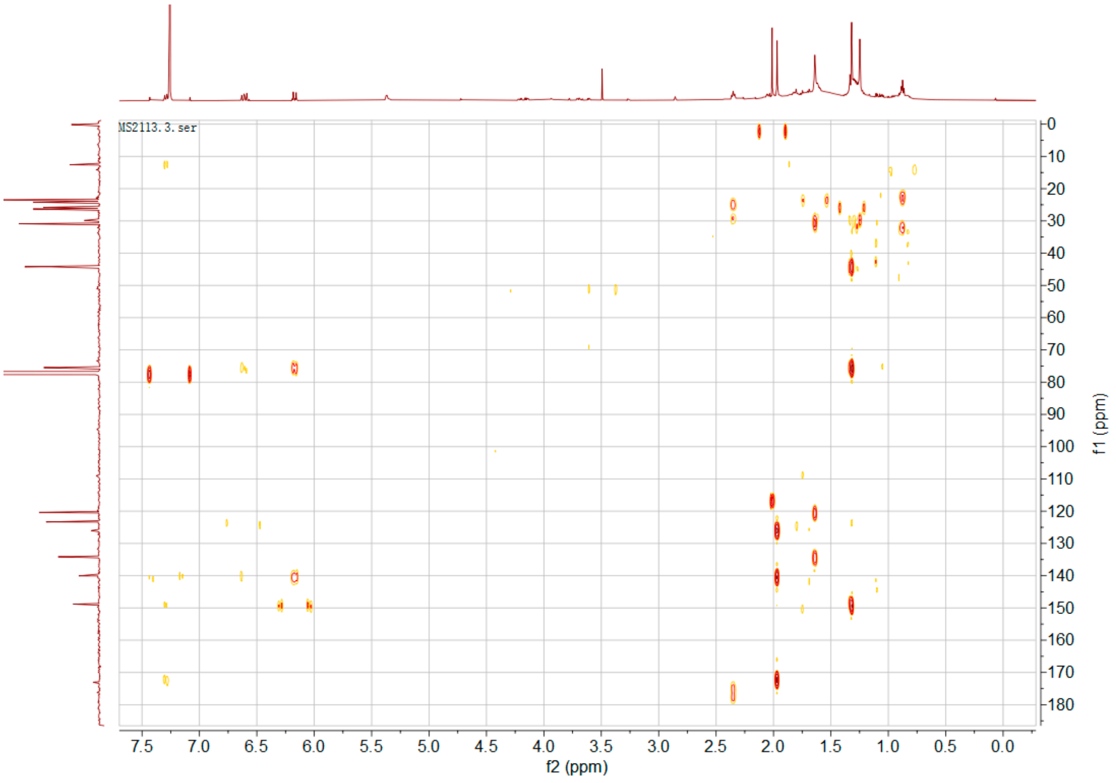

Figure S16. HMBC spectrum of elgonene N (**2**) in $CDCl_3$

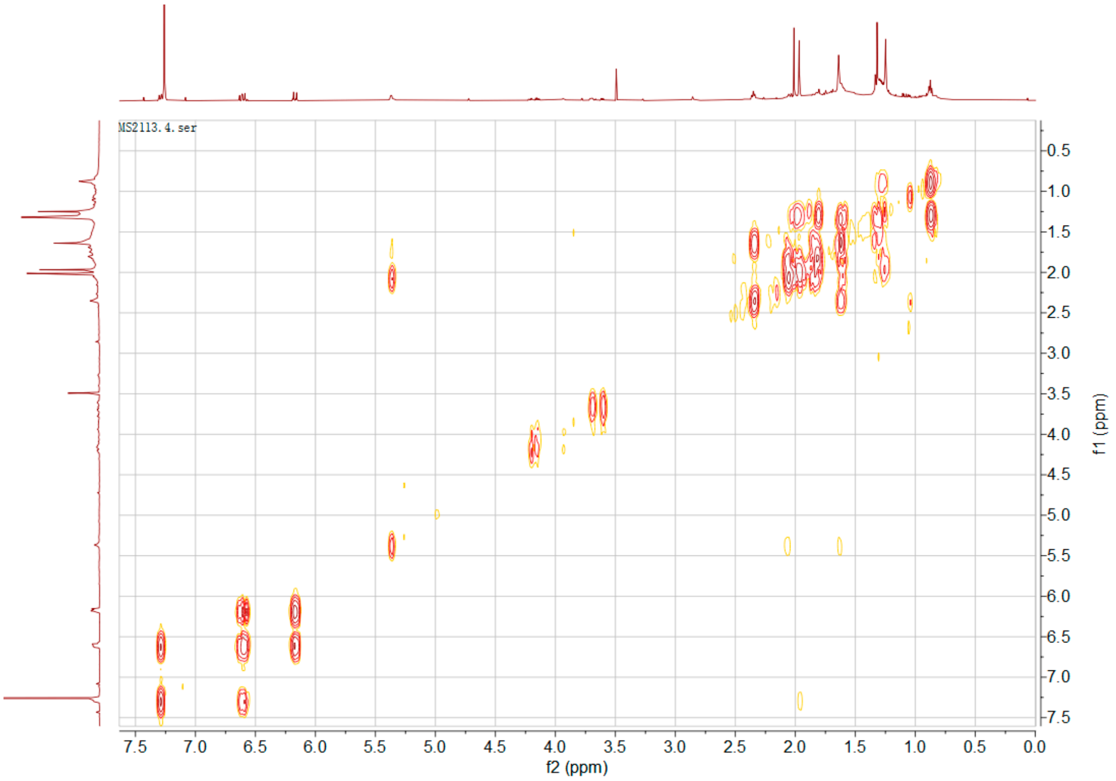

Figure S17. ¹H-¹H COSY spectrum of elgonene N (**2**) in $CDCl_3$

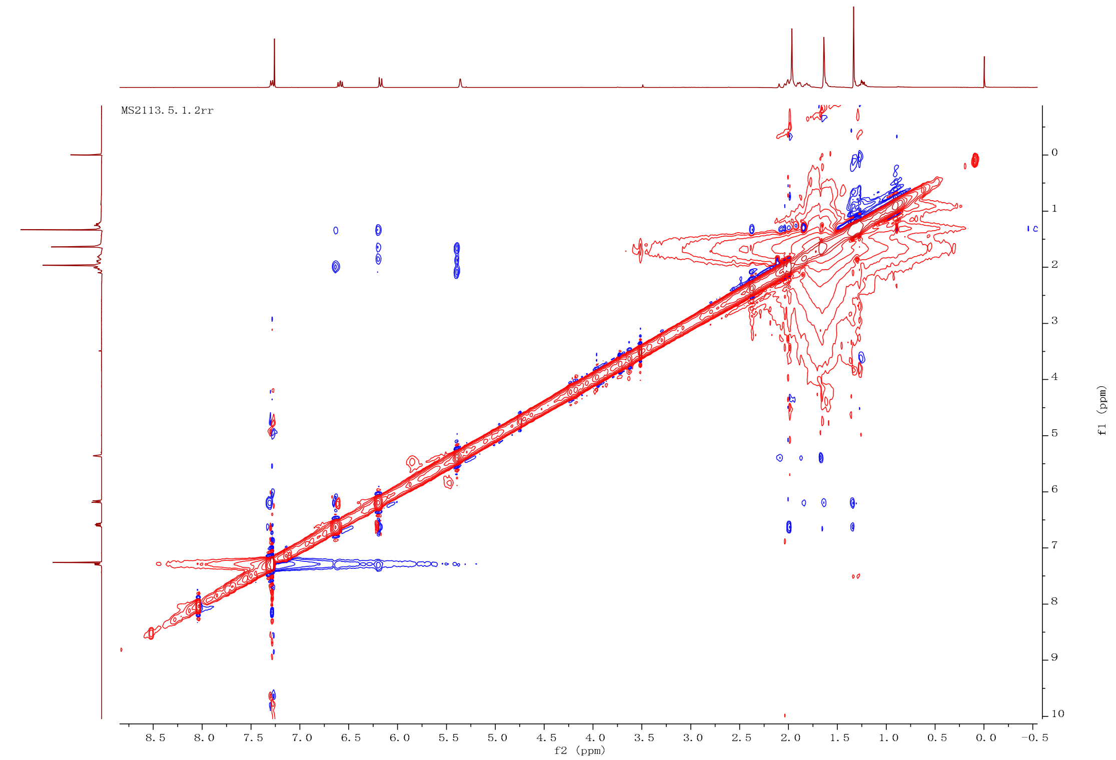

Figure S18. NOESY spectrum of elgonene N (**2**) in $CDCl_3$

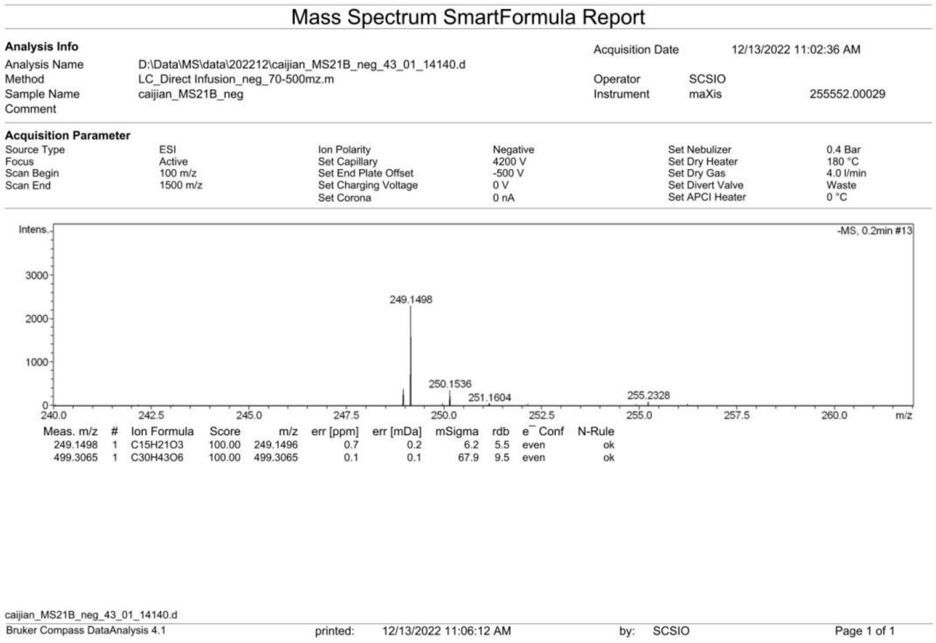

Figure S19. HRESIMS spectrum of elgonene N (**2**)

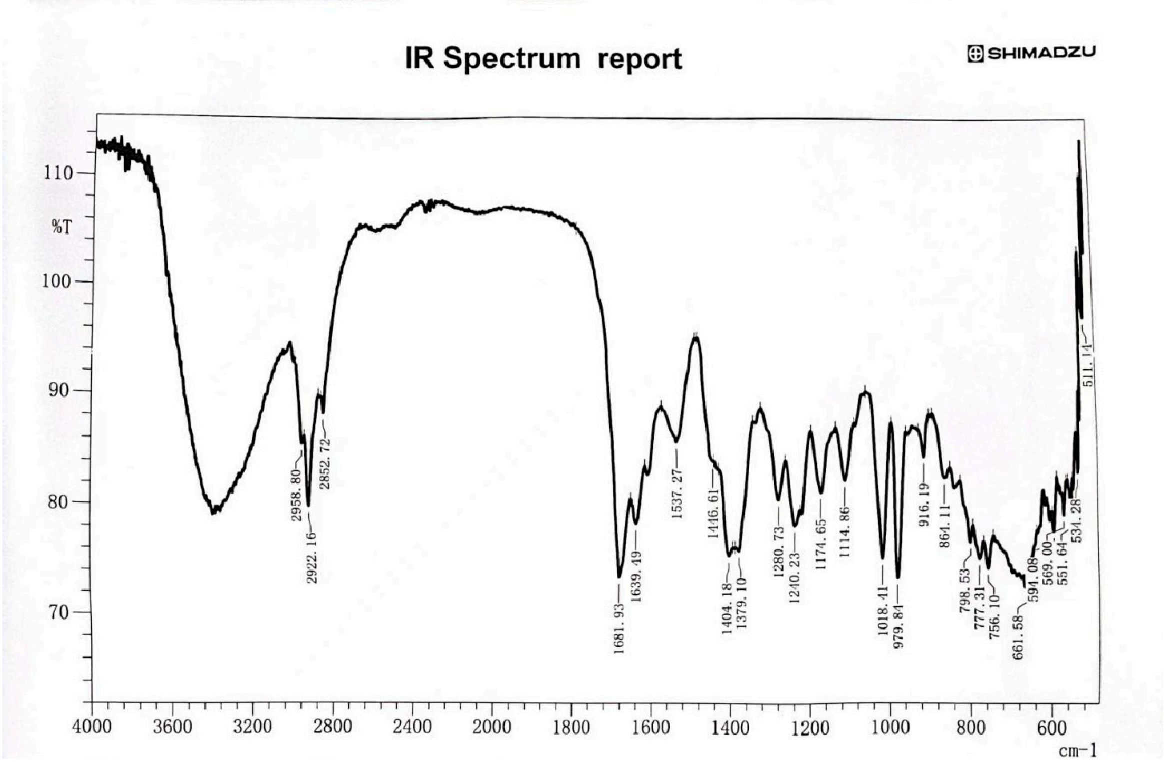

Figure S20. IR spectrum of elgonene N (**2**)

Fixed Wavelength Report Date 2007-9-2 Time 21:33:46 Page 1 of 1

Method file : <untitled>
Information : Default Method
Data File : <untitled>

Overlaid Spectra:

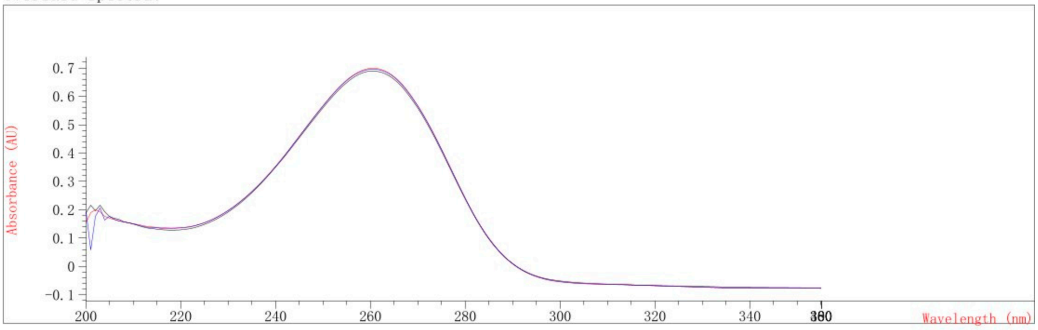

#	Name	Abs<260nm>	#	Name	Abs<260nm>	#	Name	Abs<260nm>
1		0.68990	2		0.69920	3		0.69689

Report generated by : 1 Signature: ....

*** End Fixed Wavelength Report ***

Figure S21. UV spectrum of elgonene N (**2**) in MeOH

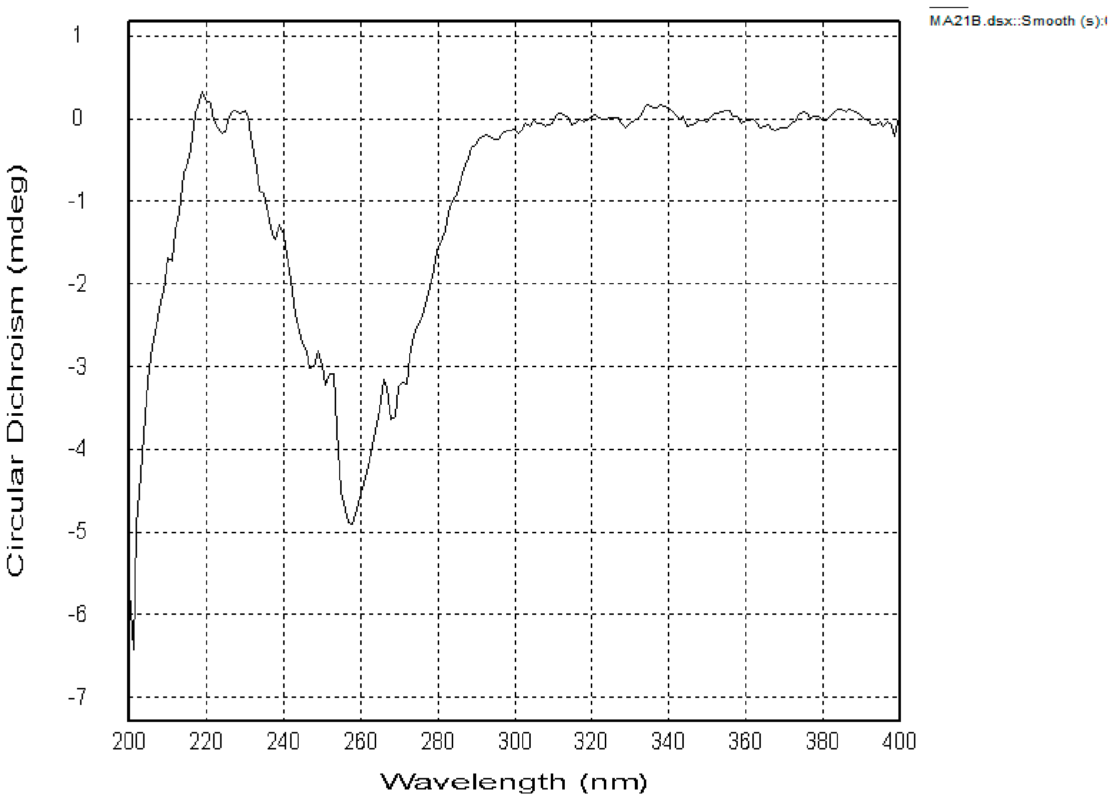

Figure S22. ECD spectrum of elgonene N (**2**) in MeOH

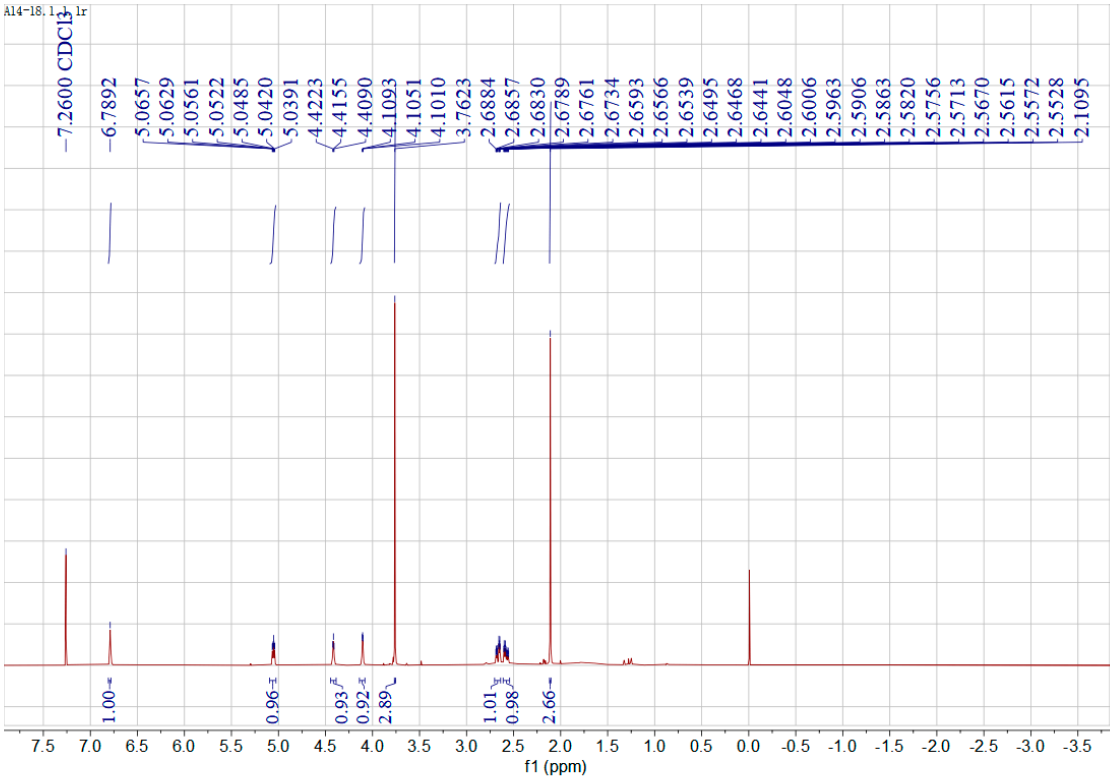

Figure S23. ^{1}H NMR spectrum of compound **3** in $CDCl_3$

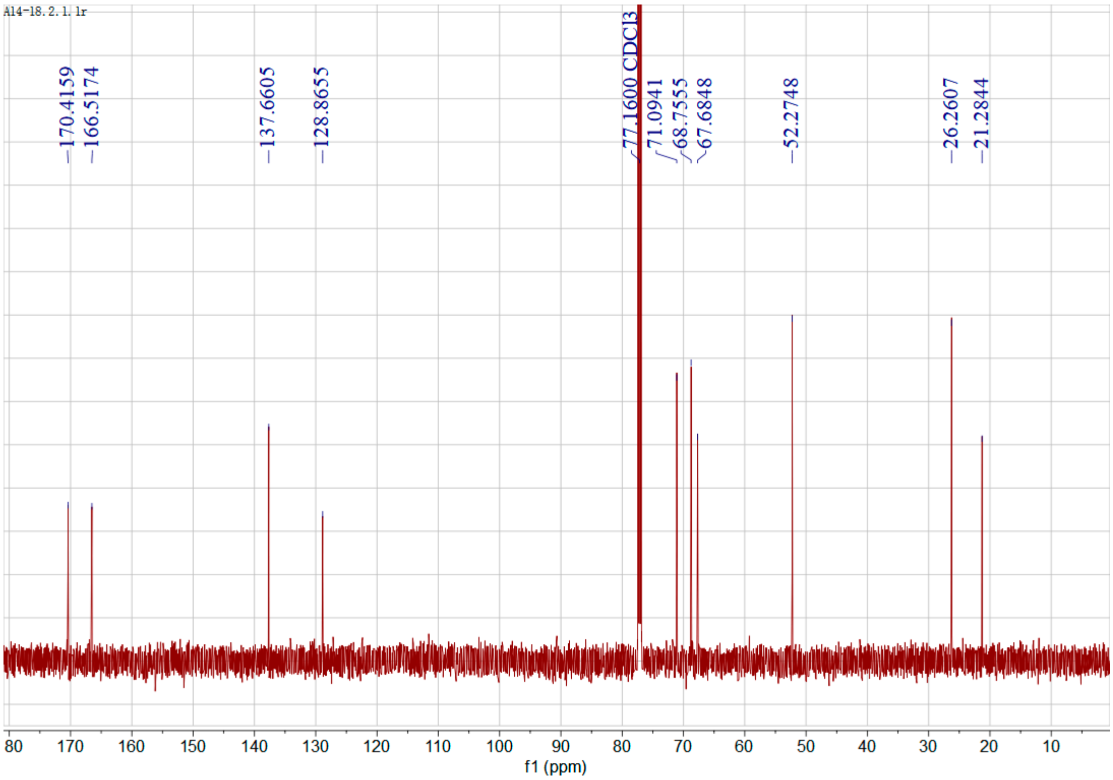

Figure S24. ^{13}C NMR spectrum of compound **3** in $CDCl_3$

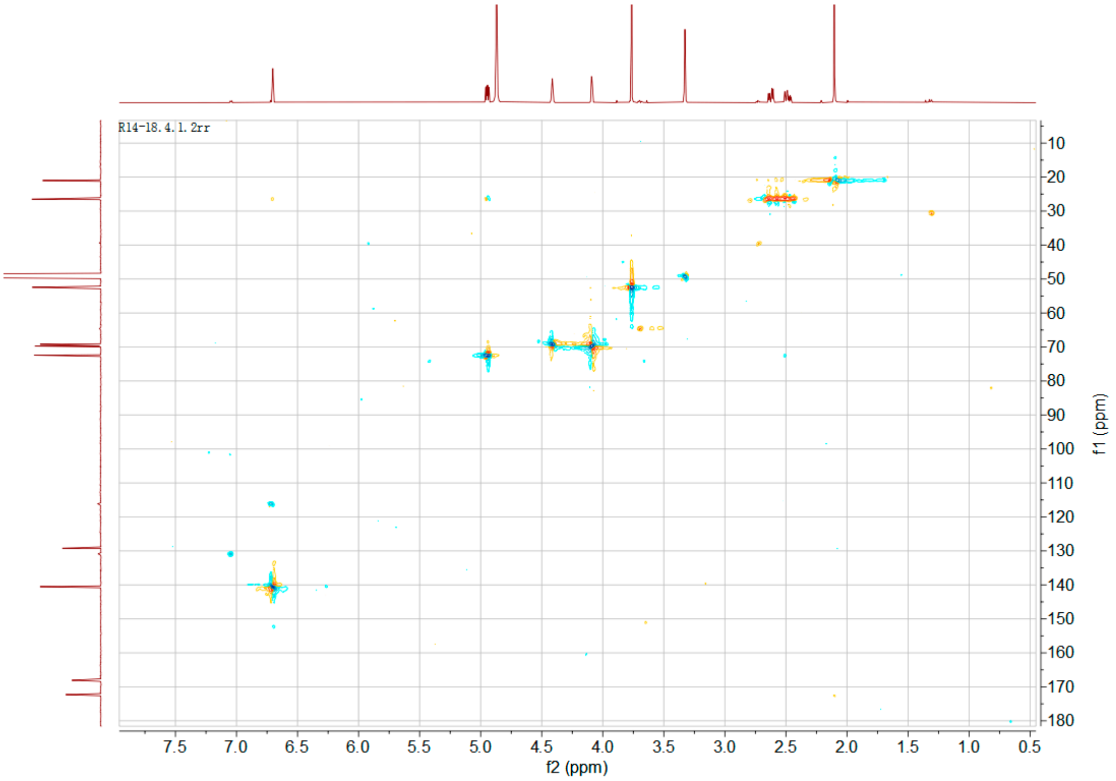

Figure S25. HSQC spectrum of compound **3** in CD_3OD

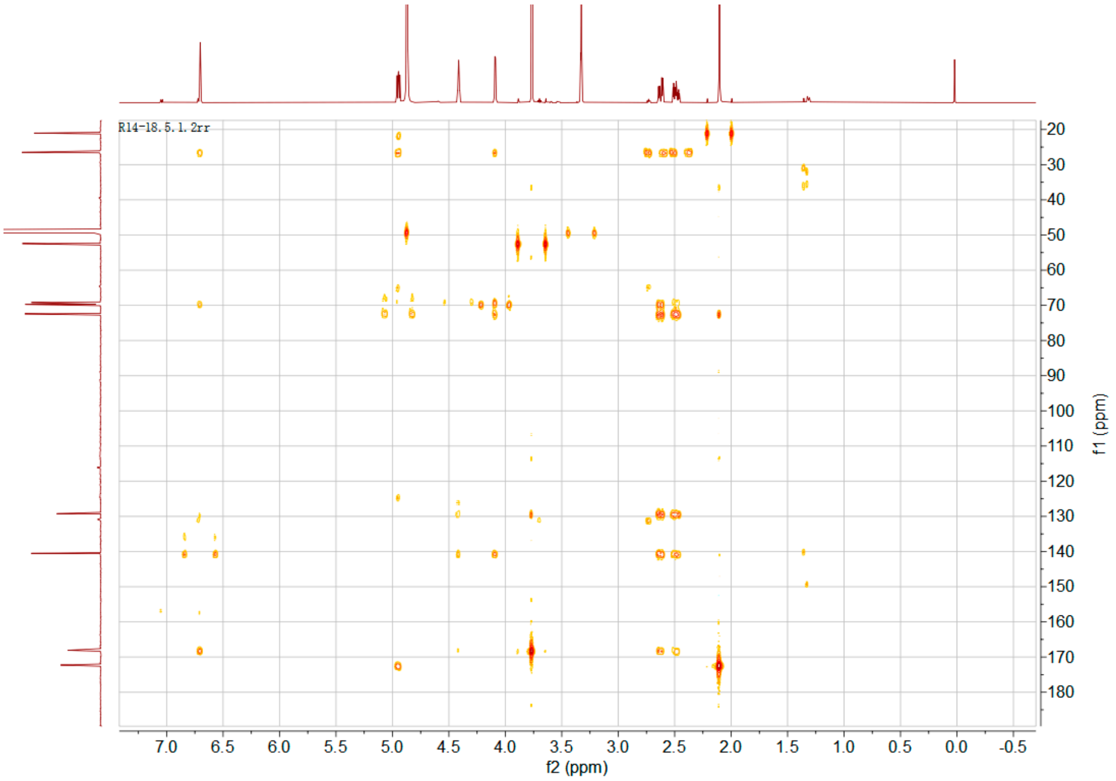

Figure S26. HMBC spectrum of compound **3** in CD_3OD

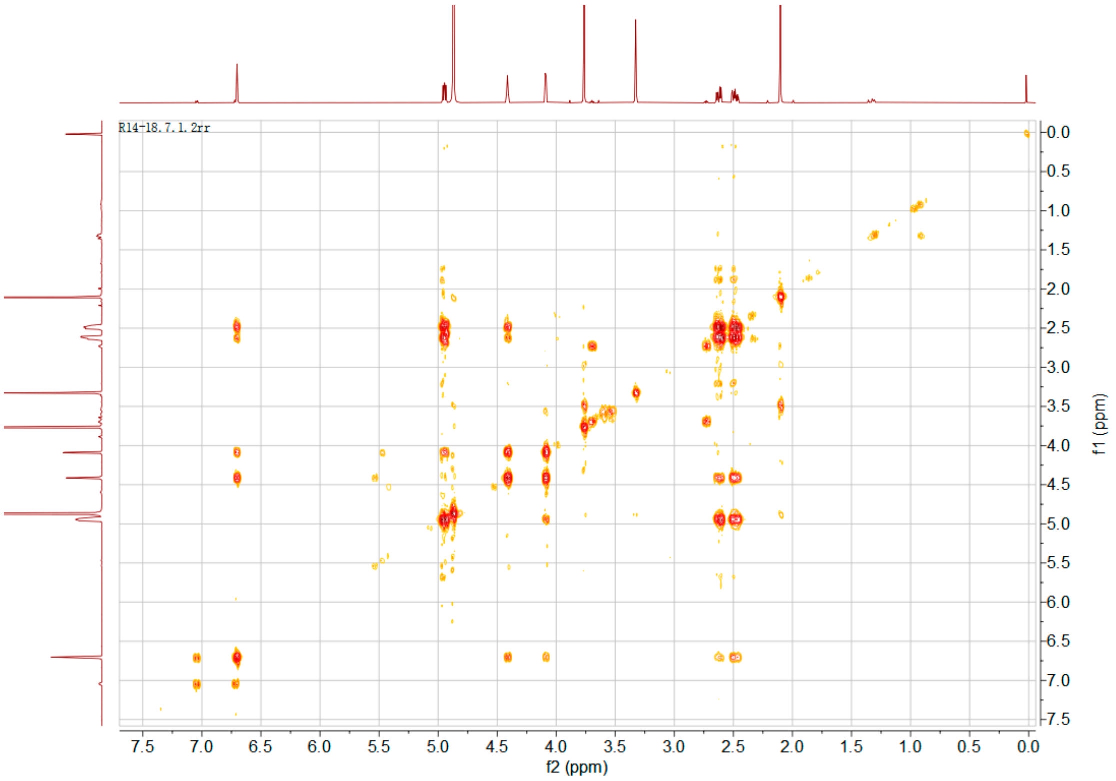

Figure S27. ^1H-^1H COSY spectrum of compound **3** in CD_3OD

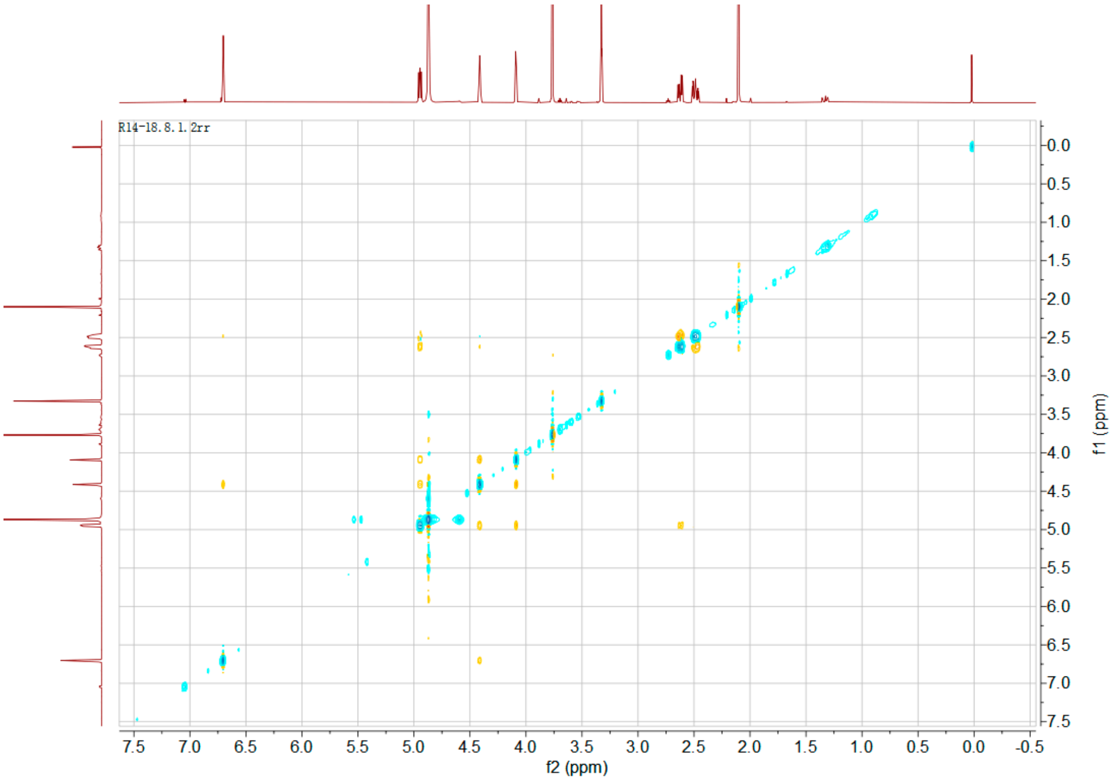

Figure S28. NOESY spectrum of compound **3** in CD_3OD

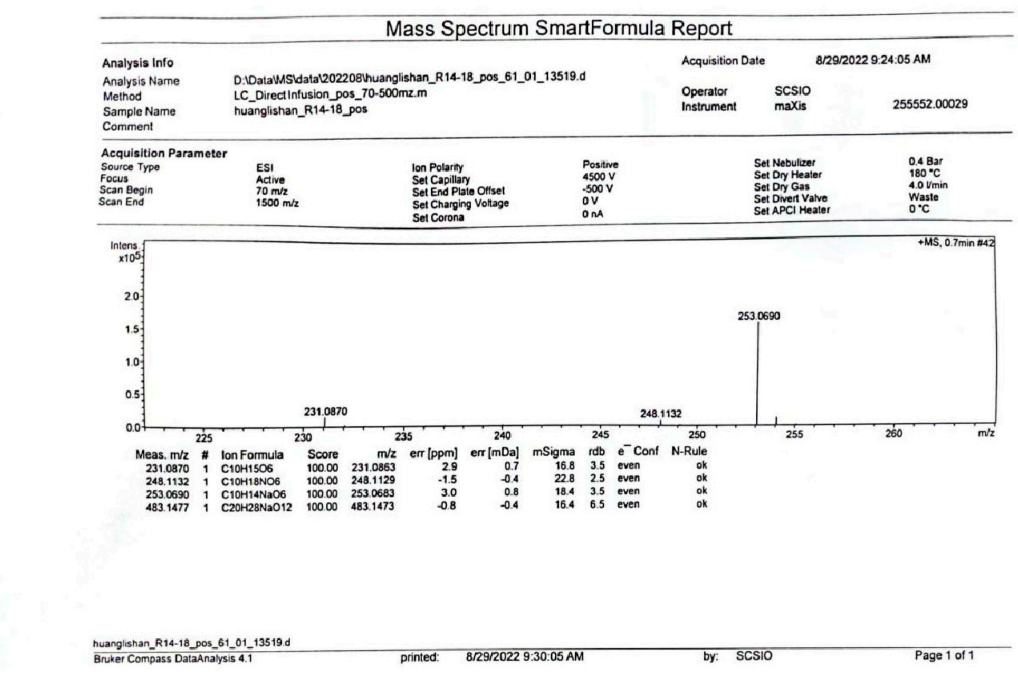

Mass Spectrum SmartFormula Report

Analysis Info		Acquisition Date	8/29/2022 9:24:05 AM
Analysis Name	D:\Data\MS\data\202208\huanglishan_R14-18_pos_61_01_13519.d	Operator	SCSIO
Method	LC_DirectInfusion_pos_70-500mz.m	Instrument	maXis 255552.00029
Sample Name	huanglishan_R14-18_pos		
Comment			

Acquisition Parameter

Source Type	ESI	Ion Polarity	Positive	Set Nebulizer	0.4 Bar
Focus	Active	Set Capillary	4500 V	Set Dry Heater	180 °C
Scan Begin	70 m/z	Set End Plate Offset	-500 V	Set Dry Gas	4.0 l/min
Scan End	1500 m/z	Set Charging Voltage	0 V	Set Divert Valve	Waste
		Set Corona	0 nA	Set APCI Heater	0 °C

Meas. m/z	#	Ion Formula	Score	m/z	err [ppm]	err [mDa]	mSigma	rdb	e⁻ Conf	N-Rule
231.0870	1	C10H15O6	100.00	231.0863	2.9	0.7	16.8	3.5	even	ok
248.1132	1	C10H18NO6	100.00	248.1129	-1.5	-0.4	22.8	2.5	even	ok
253.0690	1	C10H14NaO6	100.00	253.0683	3.0	0.8	18.4	3.5	even	ok
483.1477	1	C20H28NaO12	100.00	483.1473	-0.8	-0.4	16.4	6.5	even	ok

huanglishan_R14-18_pos_61_01_13519.d
Bruker Compass DataAnalysis 4.1 printed: 8/29/2022 9:30:05 AM by: SCSIO Page 1 of 1

Figure S29. HRESIMS spectrum of compound **3**

Fixed Wavelength Report Date 2007-9-2 Time 21:50:42 Page 1 of 1

Method file : <untitled>
Information : Default Method
Data File : <untitled>

Overlaid Spectra:

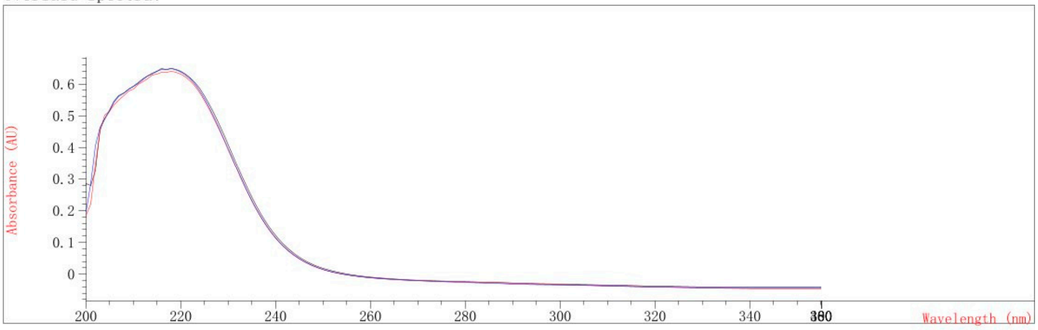

#	Name	Abs<218nm>	#	Name	Abs<218nm>	#	Name	Abs<218nm>
1		0.64852	2		0.63975	3		0.64947

Report generated by : 1 Signature: ....

*** End Fixed Wavelength Report ***

Figure S30. UV spectrum of compound **3** in MeOH

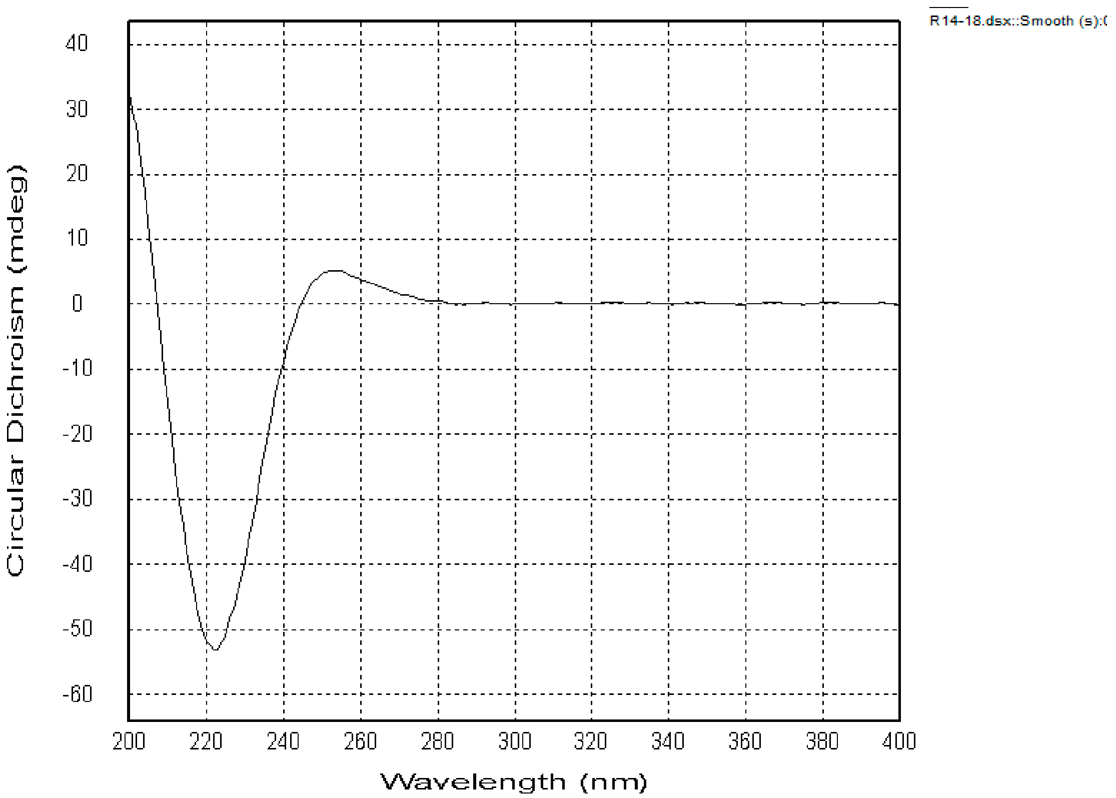

Figure S31. ECD spectrum of compound **3** in MeOH

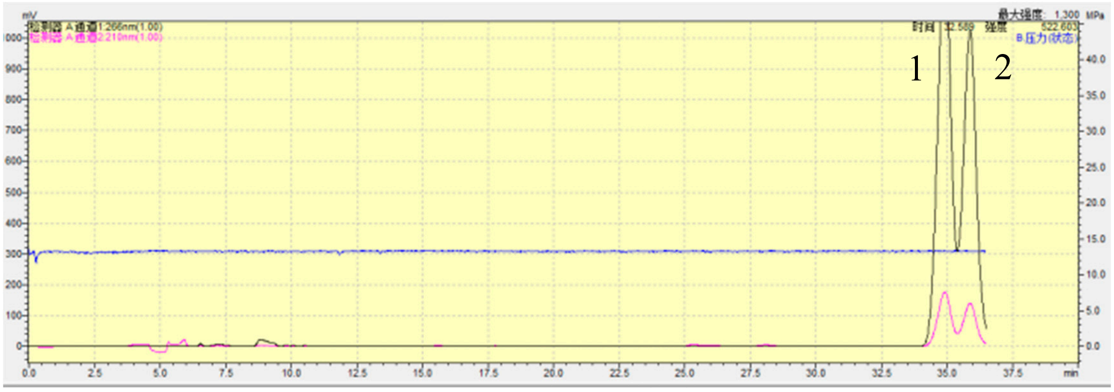

Figure S32. HPLC spectrum of compounds **1** and **2**

Table S1. Energies of **1**/**2** at B3LYP/6–311g (d, p) level.

Configuration	Conformer	E (Hartree)	E (kcal/mol)	Population
6*S*, 7*R*-1/**2**	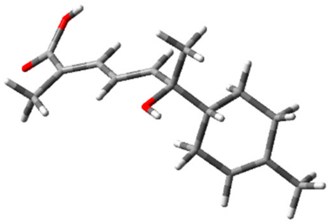	-810.576929930	-508645.129300374	6.35%

6*S*, 7*R*-1/**2**	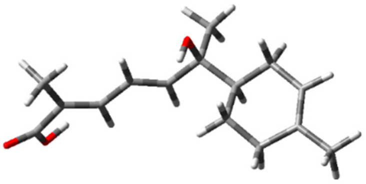	-810.575624333	-508644.310025201	1.59%
6*S*, 7*R*-1/**2**	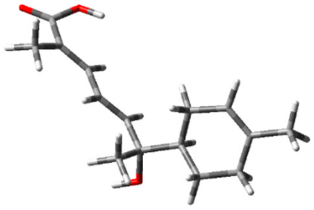	-810.576783344	-508645.037316193	5.44%
6*S*, 7*R*-1/**2**	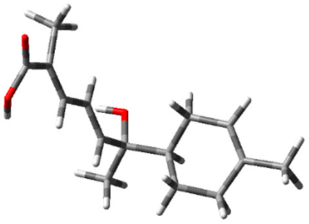	-810.579226150	-508646.570201386	72.32%
6*S*, 7*R*-1/**2**	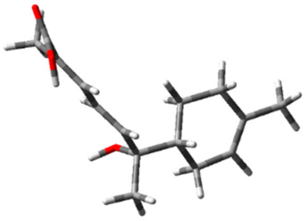	-810.576684109	-508644.975045239	4.90%
6*S*, 7*R*-1/**2**	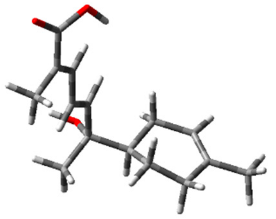	-810.577298275	-508645.360440545	9.39%
6*R*, 7*S*-1/**2**	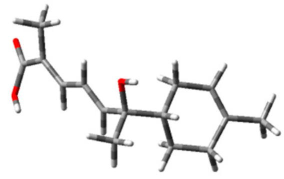	-810.576929930	-508645.129300374	6.36%
6*R*, 7*S*-1/**2**	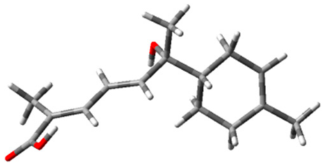	-810.575620754	-508644.307779343	1.59%
6*R*, 7*S*-1/**2**	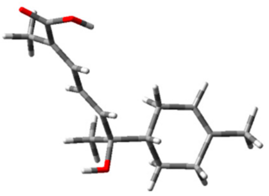	-810.576783343	-508645.037315566	5.44%

6*R*, 7*S*-1/**2**	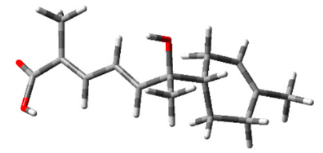	-810.579226149	-508646.570200759	72.34%
6*R*, 7*S*-1/**2**	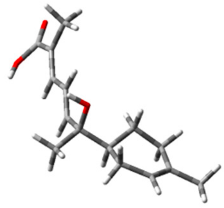	-810.576679928	-508644.972421619	4.88%
6*R*, 7*S*-1/**2**	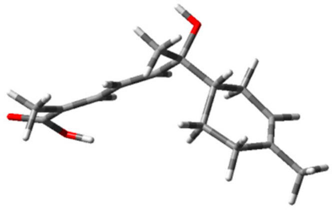	-810.577298273	-508645.360439290	9.39%
6*S*, 7*S*-1/**2**	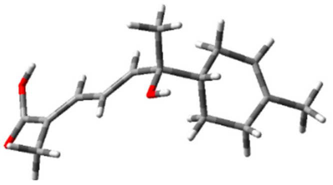	-810.574393125	-508643.537429869	1.12%
6*S*, 7*S*-1/**2**	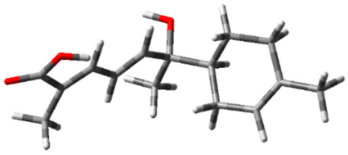	-810.578173993	-508645.909962347	61.34%
6*S*, 7*S*-1/**2**	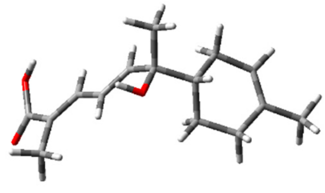	-810.576942782	-508645.137365133	16.65%
6*S*, 7*S*-1/**2**	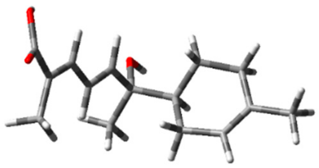	-810.576223625	-508644.686086924	7.77%
6*S*, 7*S*-1/**2**	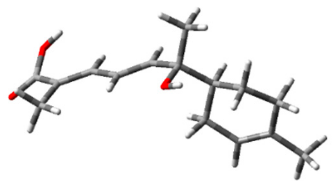	-810.575957880	-508644.519329279	5.87%
6*S*, 7*S*-1/**2**	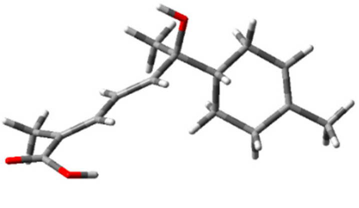	-810.576158272	-508644.645077263	7.25%

6*R*, 7*R*-1/**2**	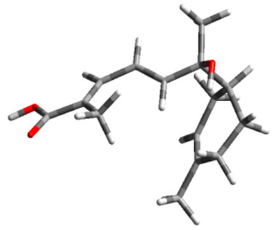	-810.576254660	-508644.705561697	5.91%
6*R*, 7*R*-1/**2**	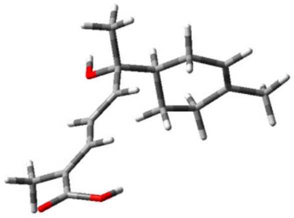	-810.574377462	-508643.527601180	0.81%
6*R*, 7*R*-1/**2**	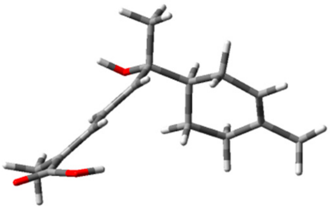	-810.576792747	-508645.043216670	10.44%
6*R*, 7*R*-1/**2**	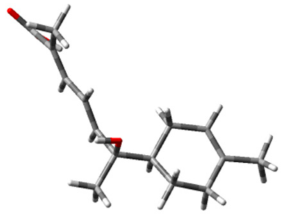	-810.577394560	-508645.420860346	19.76%
6*R*, 7*R*-1/**2**	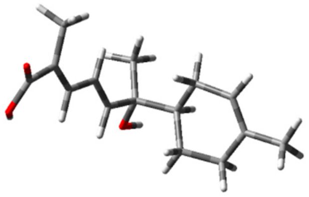	-810.576223626	-508644.686087551	5.72%
6*R*, 7*R*-1/**2**	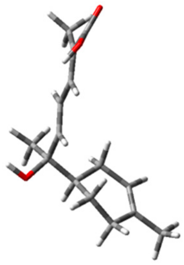	-810.578401055	-508646.052446023	57.37%

Table S2. Energies of **3** at B3LYP/6–311g (d, p) level.

Configuration	Conformer	E (Hartree)	E (kcal/mol)	Population
3*R*, 4*R*, 5*S*-**3**	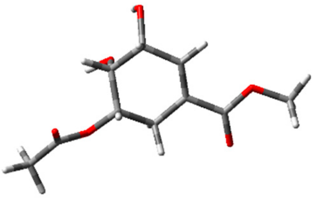	-840.919556213	-527685.430719220	50.67%

3*R*, 4*R*, 5*S*-**3**	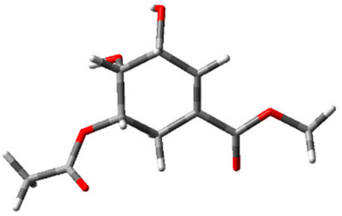	-840.919385679	-527685.323707429	42.29%
3*R*, 4*R*, 5*S*-**3**	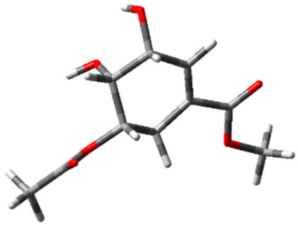	-840.915512148	-527682.893027991	0.70%
3*R*, 4*R*, 5*S*-**3**	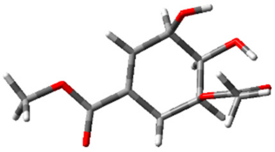	-840.917422155	-527684.091576484	5.29%
3*R*, 4*R*, 5*S*-**3**	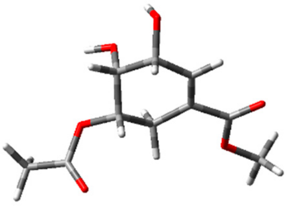	-840.915902521	-527683.137990953	1.06%
3*S*, 4*S*, 5*R*-**3**	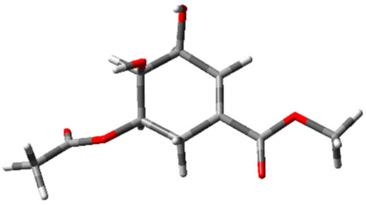	-840.919556213	-527685.430719220	50.67%
3*S*, 4*S*, 5*R*-**3**	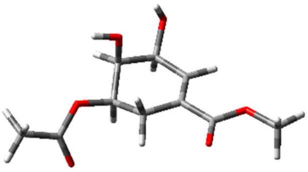	-840.919385679	-527685.323707429	42.29%
3*S*, 4*S*, 5*R*-**3**	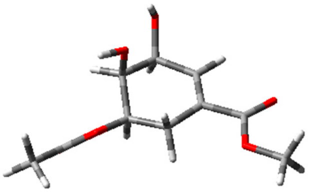	-840.915512148	-527682.893027991	0.70%
3*S*, 4*S*, 5*R*-**3**	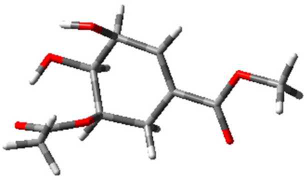	-840.917422155	-527684.091576484	5.29%

3*S*, 4*S*, 5*R*-**3**	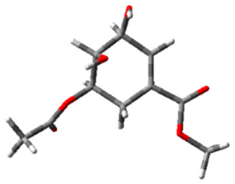	-840.915902522	-527683.137991580	1.06%
